# Trikoramides B–D, Bioactive Cyanobactins from the Marine Cyanobacterium *Symploca hydnoides*

**DOI:** 10.3390/md19100548

**Published:** 2021-09-28

**Authors:** Ma Yadanar Phyo, Teo Min Ben Goh, Jun Xian Goh, Lik Tong Tan

**Affiliations:** Natural Sciences and Science Education, National Institute of Education, Nanyang Technological University, 1 Nanyang Walk, Singapore 637616, Singapore; zaara.yadanarphyo@gmail.com (M.Y.P.); bengoh93@yahoo.com.sg (T.M.B.G.); junxiangoh@hotmail.com (J.X.G.)

**Keywords:** *Symploca hydnoides*, microcoleaceae, cyanobactin, trikoramides, cytotoxic, quorum-sensing inhibitors

## Abstract

Three new cyanobactins, trikoramides B (**1**)–D (**3**), have been isolated from the marine cyanobacterium, *Symploca hydnoides*, following a preliminary bioassay-guided isolation of the two most active polar fractions based on the brine shrimp toxicity assay. These new cyanobactins are new analogues of the previously reported cytotoxic trikoramide A (**4**) with differences mainly in the *C*-prenylated cyclotryptophan unit. Their planar structures were elucidated from their 1D and 2D NMR spectral data in combination with the HRMS/MS data. Marfey’s method, 2D NOESY NMR spectroscopic and ECD spectra analyses were used to determine the absolute stereochemistry of trikoramides B (**1**)–D (**3**). Trikoramides B (**1**) and D (**3**) exhibited cytotoxicity against MOLT-4 acute lymphoblastic leukemia cell line with IC_50_ values of 5.2 µM and 4.7 µM, respectively. Compounds **1** and **3** were also evaluated for their quorum-sensing inhibitory assay based on the *Pseudomonas aeruginosa* PAO1 *lasB-gfp* and *rhlA-gfp* bioreporter strains. Although trikoramide B (**1**) exhibited weak quorum-sensing inhibitory activity, the Br-containing trikoramide D (**3**) exhibited moderate to significant dose-dependent quorum-sensing inhibitory activities against PAO1 *lasB-gpf* and *rhlA-gfp* bioreporter strains with IC_50_ values of 19.6 µM and 7.3 µM, respectively.

## 1. Introduction

Filamentous cyanobacteria (blue-green microalgae) are oxygen-producing photoautotrophic prokaryotes whose existence dates back to 3.5 billion years ago [1]. These ancient microbes are also known to be prolific producers of novel bioactive natural products. In particular, the order *Oscillatoriales* accounts to produce most of the isolated bioactive secondary metabolites from marine cyanobacteria, most of which are cyclic or linear peptides [2]. These peptides are assembled by either the nonribosomal or post-translationally modified ribosomal biosynthetic pathways in cyanobacteria. The first ribosomal biosynthetic pathway was described by Schmidt et al. in 2005 for the cyanobactin, patellamide [3]. Cyanobactins are a class of ribosomally synthesized cyclic peptides with post-translational modifications, including formation of azole/azoline rings, D-stereocentres and prenyl group [4]. Several cyanobactins have been found to have anticancer properties (e.g., trunkamide [5]), multidrug resistance-reversing activity (e.g., dendroamides [6]) and antimalarial activity (e.g., venturamides [7]).

Previous efforts to hunt for novel bioactive secondary metabolites in our laboratory has led to the isolation of the cytotoxic prenylated cyanobactin, trikoramide A (**4**) [8] from *Symploca hydnoides*. Further investigations into the brine shrimp toxic polar fractions have afforded three new related compounds, trikoramides B (**1**)–D (**3**). Both trikoramides B (**1**) and D (**3**) exhibited significant cytotoxic activity against MOLT-4 acute lymphoblastic leukemia cell line. Interestingly, the Br-containing trikoramide D (**3**) exhibited significant quorum-sensing inhibitory (QSI) activities in a dose-dependent manner when tested in the *Pseudomonas aeruginosa* PAO1 *lasB-gpf* and *rhlA-gfp* bioreporter strains. The current study details the purification, structural determination and biological activities of these new trikoramide A related analogues.

## 2. Results and Discussions

### 2.1. Isolation and Structural Elucidation

**Figure 1:**
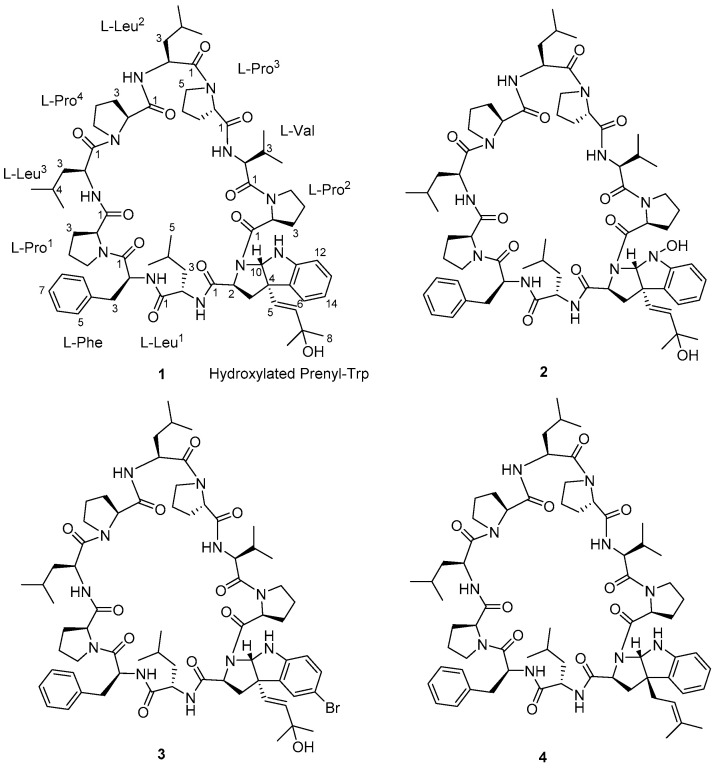
Structures of trikoramides B (**1**)–D (**3**) and A (**4**).

The marine cyanobacterium *S. hydnoides* was collected by hand at the intertidal shores of Trikora beach, Bintan Island, Indonesia. The sample was extracted exhaustively with CH_2_Cl_2_-MeOH (2:1, *v*/*v*) and the resulting crude extract was fractionated into nine fractions (1 to 9) using vacuum liquid chromatography (VLC). Fractions 8 and 9, eluted with EtOAc-MeOH (9:1) and EtOAc-MeOH (8:2), respectively, exhibited brine shrimp toxicity with at least 80% lethality when tested at 100 ppm and were further subjected to fractionation by reversed-phase solid-phase extraction (RP-SPE), followed by a series of reversed-phase high performance liquid chromatography (HPLC) purifications to yield white amorphous solids of trikoramide B (**1**, 1.1 mg), trikoramide C (**2**, 0.3 mg) and trikoramide D (**3**, 1.2 mg) (Figure 1).

**Figure 2:**
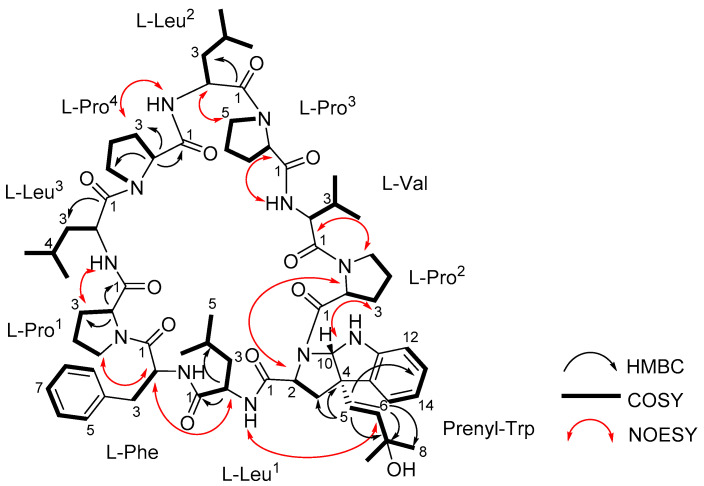
Structure of trikoramide B (**1**) with key 2D NMR correlations.

Trikoramide B (**1**) yielded a [M + H]^+^ ion at *m*/*z* 1244.7446 by HR-ESI-OrbitrapMS and is consistent with the molecular formula of C_68_H_97_N_11_O_11_ requiring 26 degrees of unsaturation. The ^1^H NMR spectrum of **1** showed signals typical of a peptidic metabolite with the *N*H signals observed between δ_H_ 6.67–δ_H_ 8.02 and α methine protons between δ_H_ 3.07–δ_H_ 4.75 (Table 1). Initial analysis of the ^13^C NMR spectrum revealed ten signals characteristic of amide carbonyl groups (δ_C_ 170.1, 172.5, 171.7, 172.9, 171.5, 170.5, 170.8, 172.9, 170.3, 170.7) indicating **1** to be a decapeptide as well as an analogue of trikoramide A (**4**) (Table 1) [8]. Further analysis of the 1D and 2D NMR spectra of **1** established the presence of nine standard amino acids, including 4 × Pro, 1 × Leu, 1 × Val and 1 × Phe, and an atypical amino acid. The identity of the unusual amino acid was determined to be a hydroxylated *C*-prenylated-cyclotryptophan by a combination of ^13^C, ^1^H, COSY, HSQC and HMBC NMR spectra which have been interpreted as follows. From the ^13^C and DEPT-135 NMR spectra, we observed the presence of four methine carbons (δ_C_ 110.5, 119.2, 123.7, 129.3) and two non-protonated aromatic carbons (δ_C_ 130.6, 149.2) characteristic of an indoline moiety, which was also evident from the UV absorption at λ_max_ 250 and 295 nm, similar to that of the previously reported trikoramide A (**4**) [8]. However, the chemical shifts of the olefinic carbons at the prenyl unit in **1** shifted downfield from δ 118.3/5.14 (H-6) and δ 135.8 (C-7) in trikoramide A (**4**) to δ 126.7/5.81 (H-5) and δ 139.1/5.52 (H-6) in **1**. In addition, based on the large coupling constant of 15.0 Hz between H-5 and H-6, these two vicinal protons are trans to each other suggesting a shift in the position of the double bond (H-5/H-6) in the prenyl unit. Furthermore, HMBC correlations between H-5 and H-6 to C-7 (δ_C_ 70.4) and presence of a dehydrated protonated molecule [M + H – H_2_O]^+^ peak at *m*/*z* 1226.7322 confirmed the presence of a tertiary alcohol with two methyl groups attached (Figure 2) at the prenyl residue. The COSY correlation between H-5 and H-6 in combination with several key HMBC correlations, including H-5/C-4, H-5/C-3, H-5/C-15, H-6/CH_3_-8, and H-6/CH_3_-9, established that the prenylated unit was attached to the indolic cyclotryptophan unit (Figure 2). This confirms the presence of a *C*-prenylated cyclotryptophan with a terminal tertiary alcohol group on the prenylated side chain.

The sequence of the amino acids in **1** was inferred from 2D NMR spectral data as well as MS/MS spectral analysis. Nine key NOESY correlations between H-2 of Phe and H-5a of Pro^1^, H-2 of Leu^1^ and H-2 of Phe, *N*-H of Leu^1^ and H-6 of Prenyl-Trp, H-2 of Prenyl-Trp and H-2 of Pro^2^, H-2 of Val and H-5a of Pro^2^, *N*-H of Val and H-2 of Pro^3^, H-2 of Leu^2^ and H-5a of Pro^3^, *N*-H of Leu^2^ and H-3a of Pro^4^, H-3a of Pro^1^ and *N*-H of Leu^3^ established the presence of the sequence Leu^3^-Pro^1^-Phe-Leu^1^-Prenyl-Trp-Pro^2^-Val-Pro^3^-Leu^2^-Pro^4^ (Figure 2). Additional evidence from the fragmentation pattern observed in the MS/MS spectrum of trikoramide B (**1**) confirms the presence of the aforementioned sequence and the double bond equivalents calculated from its molecular formula suggests the structure to be a cyclic decapeptide, enacting the structure as drawn in **1**. The geometries of the amide bonds in the proline residues were determined based on the ^13^C NMR chemical shift difference at the β and γ positions (Δδ_β−γ_). Similar to trikoramide A (**4**), the large difference calculated for Pro^1^ (Δδ_β−γ_ =8.9 ppm) and Pro^2^ (Δδ_β−γ_ =8.1 ppm) indicated that their peptide bonds were in cis geometry. For Pro^3^ (Δδ_β−γ_ =4.5 ppm) and Pro^4^ (Δδ_β−γ_ = 3.5 ppm) their geometries were determined to be trans which was also further supported by NOESY correlations between H-2 of Leu^2^ and H-5a/5b of Pro^3^; H-2 of Leu^3^ and H-5a/5b of Pro^4^ (Figure 2).

**Figure 3:**
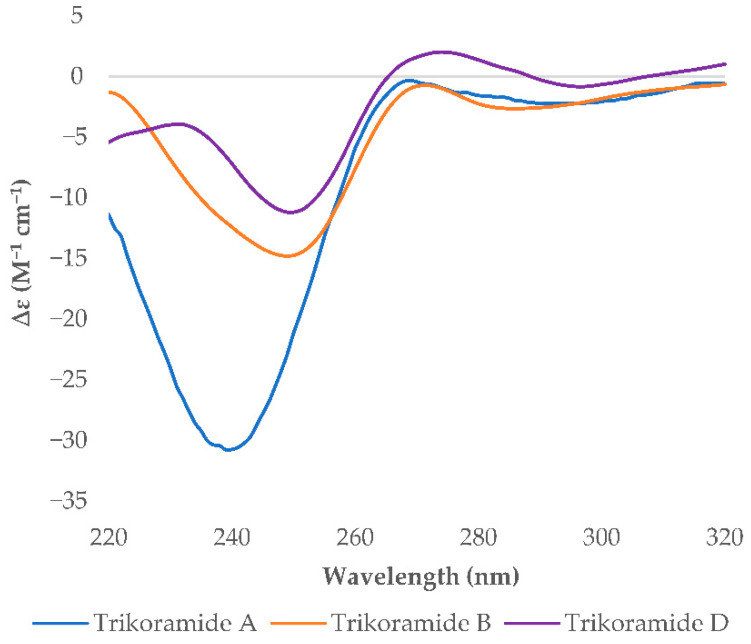
Experimental ECD spectra of trikoramides A (**4**), B (**1**) and D (**3**).

The absolute stereochemical configurations of the usual amino acids in trikoramide B (**1**) were determined by acid hydrolysis followed by derivatizing with Marfey’s reagent 1-fluoro-2,4-dinitro-phenyl-5-L-alanine amide (L-FDAA), revealing the L-configuration of Pro, Val, Leu and Phe residues [9]. For the hydroxylated Prenyl-Trp residue, acid hydrolysis of **1** with 1% phenol yielded tryptophan residue from the Prenyl-Trp residue and subsequent derivatization with Marfey’s reagent afforded L-Trp, indicating the absolute configuration of C-2 on Prenyl-Trp residue to be of *S* configuration. Analysis of the NOESY spectrum of **1** showed absence of cross peak between H-5 and H-10 of the Prenyl-Trp residue, indicating that they were on the different face of the tricyclic indole ring. This suggested that there were only two possible absolute configurations of the Prenyl-Trp unit at (2*S*, 4*S*, 10*S*) or (2*S*, 4*R*, 10*R*). The electronic circular dichroism (ECD) spectrum of trikoramide B (**1**) was investigated to determine the absolute stereochemistry at C-4 and C-10 of the Prenyl-Trp residue. The ECD spectrum of **1** was compared to the previously reported ECD spectrum of motobamide, a cyclic peptide containing similar Prenyl-Trp unit, which showed a negative Cotton effect at 295 nm [10]. Takahashi et al. designed two model compounds with the absolute stereochemistries of 2*S*, 4*S*, 10*S* and 2*S*, 4*R*, 10*R* of the Prenyl-Trp and calculated the theoretical ECD spectra of the model compounds and compared with the experimental ECD spectrum of motobamide [10]. The resulting ECD spectrum for the (2*S*, 4*S*, 10*S*) model compound exhibited negative Cotton curves similar to that of motobamide and its absolute stereochemistry was directly correlated [10]. Similarly, the ECD spectrum of trikoramide B (**1**) portrayed negative Cotton effect at 295 nm, contributed solely by the Prenyl-Trp unit, and is similar to the ECD spectra of the simplified model compound with 2*S*, 4*S*, 10*S* as well as motobamide (Figure 3). Furthermore, the ECD spectrum of the previously isolated trikoramide A (**4**) was obtained and it showed negative Cotton curves similar to that of **1**, suggesting the absolute stereochemistry of the hydroxylated Prenyl-Trp residue to be 2*S*, 4*S*, 10*S* (Figure 3).

**Figure 4:**
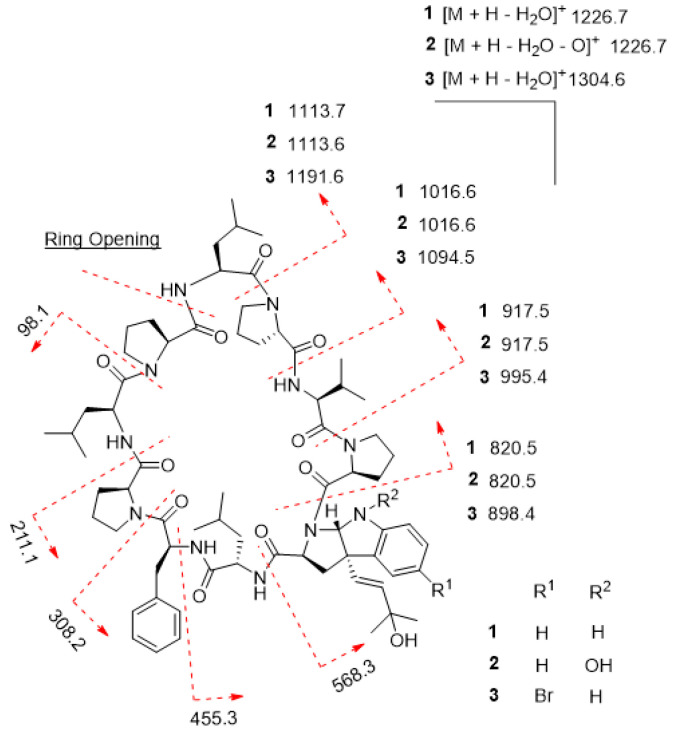
MS/MS fragmentation of trikoramides B (**1**)–D (**3**).

Trikoramide C (**2**) gave a [M + H]^+^ protonated molecule at *m*/*z* 1260.7386 by HR-ESI-OrbitrapMS, consistent with the molecular formula of C_68_H_97_N_11_O_12_ with an additional 16 mass units equating to an additional oxygen atom compared to trikoramide B (**1**). Further analysis of the ^1^H NMR spectrum of **2** in conjunction with the HR-ESI-OrbitrapMS spectrum established the presence of an unusual hydroxylamine group on the Prenyl-Trp residue. The ^1^H NMR spectrum of trikoramide C (**2**) showed similar chemical shifts to that of **1**. However, the chemical shifts of the H-2α proton and the H-10 singlet proton signals of the Prenyl-Trp group shifted downfield from δ_H_ 3.86/ δ_H_ 5.42 in **1** to δ_H_ 3.94/ δ_H_ 5.63 in **2**, indicating the presence of a nearby electronegative atom. Further evidence from the [M − H_2_O − O]^+^ peak from the HR-ESI-OrbitrapMS and the absence of NH proton signal on the Prenyl-Trp residue from ^1^H NMR spectrum confirms the substitution of a hydroxyl group on the indole nitrogen where the loss of 16 mass units was accounted for by the loss of oxide from the hydroxylamine group. Moreover, the chemical shifts, coupling constant values and MS/MS fragmentation pattern due to the hydroxylated prenyl side chain in **2** is similar to that in **1**, suggesting that the chemical nature of this prenyl side chain is retained in trikoramide C (Figure 4). As the quantity of the isolated trikoramide C (**2**) was obtained in the sub milligram range (about 0.3 mg), ^13^C signals of the quaternary carbons in the compound are mostly indiscernible due to low signal to noise ratio and hence not reported (Table 2).

A high-resolution mass spectrum of trikoramide D (**3**) yielded a [M + NH_4_${]}^{+}$ ion peak at 1339.6805 by HR-ESI-OrbitrapMS for a molecular formula of C_68_H_96_^79^BrN_11_O_11_ with an isotopic pattern indicative of a mono brominated decapeptide (Figure S3 in Supplementary Material). The ^1^H and ^13^C NMR chemical shifts of trikoramide D (**3**) are similar to that of trikoramide B (**1**) but only differs in the aromatic chemical shifts of the indole ring of the hydroxylated Prenyl-Trp residue. A substituted aromatic residue of the tricyclic moiety was immediately evident from the absence of an aromatic methine carbon C-14 (δ_C_ 119.0) in **1** which was replaced by a more relatively shielded quaternary carbon (δ_C_ 110.2). The substituent was assigned as Br and was located at meta position on the basis of HMBC (H-13/C-14, H-13/C-15) and COSY (H-12/H-13) NMR spectra. Since the ^1^H NMR spectra of trikoramides B (**1**)–D (**3**) as well as A (**4**) showed similar chemical shifts and have similar negative cotton curves in the ECD spectra, trikoramides C and D were assumed to have the same absolute configuration as **1**.

### 2.2. Biosynthetic Pathway of the Trikoramides

Cyanobactins are ribosomally synthesized and post-translationally modified peptides (RiPPs) encompassing post-translational modifications [11], such as heterocyclization (e.g., patellamide A) [12], oxidation, methylation (e.g., microcyclamide) [13] and prenylation (e.g., trunkamide) [5]. Cyanobactin biosynthesis begins with the precursor E-peptide, comprising of the N-terminal sequence recognized by the cleaving/modifying enzymes. Subsequently, the precursor peptide or recognition sequence is cleaved, leaving a free amine for macrocyclization and other transformation, such as cyclization and prenylation of tryptophan residue as in the case of trikoramides [14]. Prenylation happens in tandem with the formation of the new ring to form the tricyclic cyclotryptophan unit as evident from the studies done by Parajuli et al. on the biosynthetic pathway of kawaguchipeptins [15].

Three new cyanobactins, trikoramides B (**1**)–D (**3**) have been isolated from the marine cyanobacterium *S. hydnoides*. They are structurally related to the previously isolated trikoramide A (**4**) [8] with differences only in the *C*-prenylated cyclotryptophan residue. One could envision that trikoramides B (**1**)–D (**3**) underwent further post-translational enzymatic modifications by hydroxylase and tryptophan brominase giving rise to the unusual oxygenated isoprene, hydroxylamine and brominated indoline ring systems. Incidentally, a recent paper by Nguyen and co-workers reported the presence of broad-spectrum regiospecific peptidyl tryptophan-6-brominase on RiPP substrate. This brominase enzyme was detected through the mining for halogenating enzymes in sponge metagenomes [16]. To our knowledge, trikoramides C (**2**) and D (**3**) are the first hydroxylamine- and brominated cyclotryptophan-containing cyanobactins, respectively, from *S. hydnoides*.

### 2.3. Biological Activity of the Trikoramides

*P. aeruginosa* is a Gram-negative bacterial pathogen which causes acute and severe chronic infections, such as cystic fibrosis (CF) and diffuse panbronchiolitis (DPB) in patients [17]. There has been an increasing trend to further understand the mechanism of bacterial pathogenesis and intercellular microbial communication which has led to potential strategies to treat the widespread multidrug resistant bacterial diseases [18]. One such strategy is the uncovering of quorum-sensing (QS) system used by the bacteria to contemporise physiological activities of bacteria based on cell density through genetic manipulation [19]. The QS system involves synthesis of autoinducers (AI), such as *N*-acyl homoserine lactone, intracellularly by Gram-negative bacteria, which are actively or passively exchanged with the surrounding environment [20]. Gene expression related to the physiological activities is then triggered when the cell density is sufficient for the concentration of autoinducers to surpass the threshold [20]. Therefore, recent years has seen intensive research focusing on the interference of the QS system in pathogenic bacteria for development of novel therapeutics.

Although most of the QS inhibitory (QSI) molecules discovered to date are from marine invertebrates, such as corals and sponges, there has been reports of QS inhibitory compounds from marine cyanobacteria. These cyanobacterial QSI compounds include honaucins A–C [21], 8-epi malyngamide C [22] and lyngbyastatin 3 [23], which has motivated the investigation of QSI compounds from these microbes in our research. Several these compounds have been reported to possess significant QSI activity. For instance, extracts of a well-studied cyanobacterium *Leptolyngbya crossbyana* produced small molecular weight compounds, honaucins A–C, which exhibited dose-dependent QSI activity with IC_50_ values of 5.6, 17.6 and 14.6 µM, respectively against *Vibrio harveyi* BB120 [21]. 8-epi malyngamide C, isolated from the extracts of *Lyngbya majuscula* collected at various sites in Florida, was able to reduce 3-oxo-C_12_-HSL induced signaling in a *Las*R-based QS reporter (pSB1075) at a concentration of 10 µM [22]. QS inhibitory activities were not only limited to small molecular weight compounds but also present in larger compounds, such as peptidic lyngbyastatin 3, also isolated from *L. majuscula*, which inhibited the QS of bacterial reporter *C. violaceum* CV017 with an MIC of 12 µM [23].

**Figure 5:**
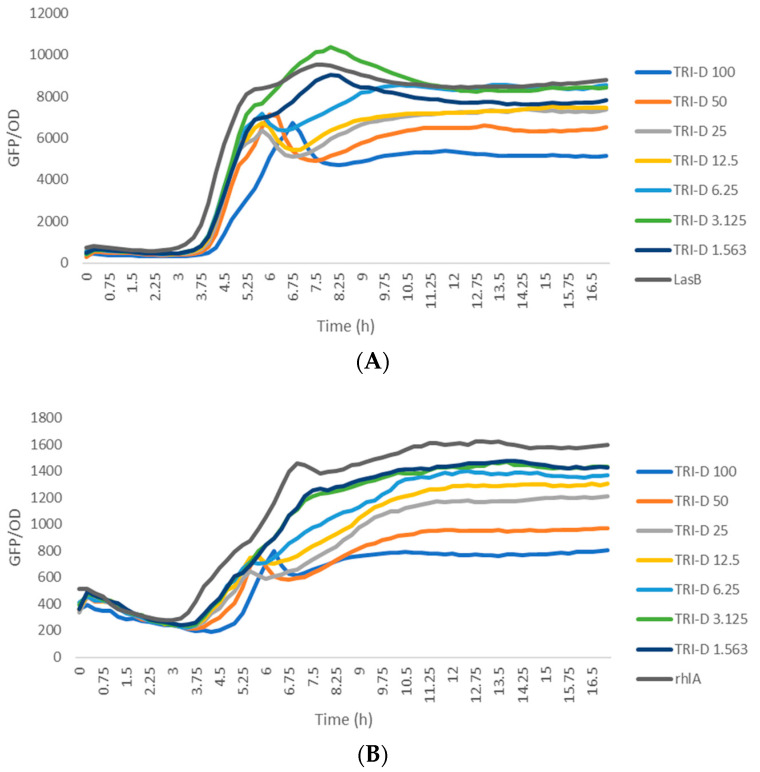
Dose response curves of trikoramide D (**3**) when incubated with *P. aeruginosa* PAO1 *lasB-gfp* (**A**) and *rhlA-gfp* (**B**) bioreporter strains.

**Figure 6:**
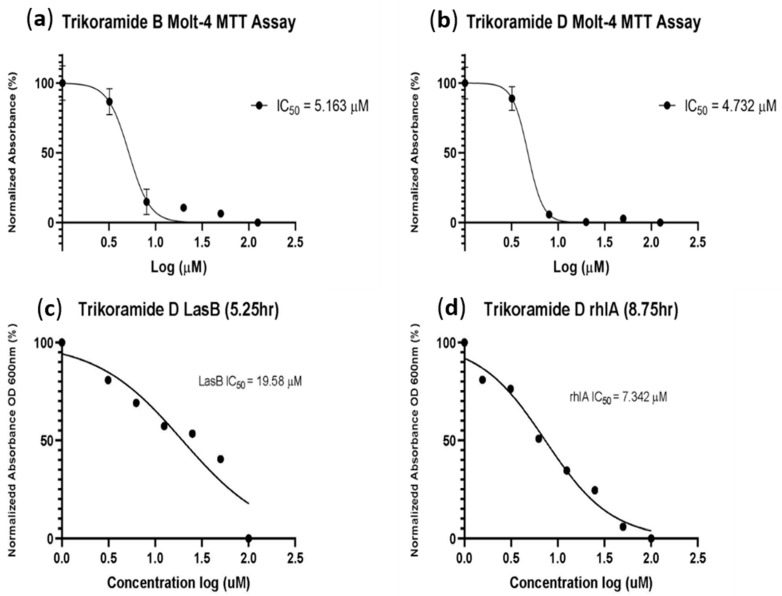
(**a**,**b**) showing log concentrations (µM) of trikoramides B (**1**) and D (**3**) against normalized absorbance (%); (**c**,**d**) for the MTT assay performed on MOLT-4 human leukemia cell line and quorum-sensing inhibitory assay based on *P. aeruginosa* PAO1 *lasB-gfp* and *rhlA-gfp* bioreporter strains.

The biological activity of trikoramide C (**2**) was not evaluated due to its minute quantity. Trikoramide B (**1**) exhibited moderate quorum-sensing inhibitory activity in assays based on *P. aeruginosa* PAO1 *lasB-gfp* and *rhlA-gfp* bioreporter strains, showing approximately 52% reduction in fluorescence when tested at an initial concentration of 100 µM. However, dose-dependent response was not observed when **1** was tested at concentrations ranging from 100 µM to 1.563 µM. Conversely, the brominated trikoramide D (**3**) exhibited significant QS inhibition in both PAO1 *lasB-gfp* and *rhlA-gfp* bioreporter strains in a dose-dependent manner (Figure 5) with low micromolar IC_50_ values of 19.6 µM and 7.3 µM, respectively (Table 3 and Figure 6). The higher potency of **3** could potentially be contributed by the brominated indole ring at the hydroxylated Prenyl-Trp residue.

Trikoramide B (**1**) and D (**3**) were also investigated for their cytotoxic activity based on the MTT assay using MOLT-4 acute lymphoblastic leukemia cell line (Table 3 and Figure 6). Both compounds exhibited similar significant cytotoxic activities, with IC_50_ values of trikoramides B and D at 5.2 µM and 4.7 µM, respectively (Table 3 and Figure 6). In addition to the trikoramides, a recently reported *C*-prenylated cyclotryptophan-containing cyanobactin, motobamide, was reported from *Leptolyngbya* sp. with antitrypanosomal property but with weak cytotoxicity activity [10].

## 3. Experimental

### 3.1. General Experimental Procedures

Optical rotations were measured on Anton Paar Polarimeter while UV and IR spectral readings were measured on a PerkinElmer UV-Visible spectrophotometer and a PerkinElmer spectrum 100 FT-IR spectrophotometer, respectively. ECD spectra were recorded on a ChirascanTM circular dichroism spectrometer. All NMR spectra were recorded in CDCl_3_ on a 400 MHz Bruker NMR Spectrometer (400.13 MHz ^1^H, 100.61 MHz ^13^C) using residual solvent signals as internal references (referenced to residual CDCl_3_ observed at δ_H_ 7.24 or δ_C_ 77.0) with chemical shifts given in ppm downfield from TMS. Isolation and purification of the trikoramides were conducted on Shimadzu LC-8A preparative LC coupled to a Shimadzu SPD-M10A VP diode array detector HPLC. High-resolution MS data and MS/MS data were acquired on Q ExactiveTM Plus Hybrid Quadrupole-Orbitrap Mass Spectrometer (Thermo Fisher Scientific, Waltham, MA, USA) equipped with a heated electrospray ionization (H-ESI) probe.

### 3.2. Sample Collection

Marine cyanobacterial samples, with cell morphology resembling that of *Symploca hydnoides* Kützing ex Gomont 1892 (Microcoleaceae), was collected in April 2018 by hand from the intertidal shores of Trikora beach, Bintan Island (GPS 1.1546879, 104.5783424). Samples were subsequently stored in 70% EtOH at −20 °C before workup at NIE. The cyanobacterial sample was subsequently confirmed as *S. hydnoides* based on phylogenetic analysis in a previous study [10]. The voucher specimen, TLT/Tri/22Apr2018/001, is deposited at Natural Sciences and Science Education, National Institute of Education, Singapore.

### 3.3. Extraction and Isolation of Compounds

Marine cyanobacterial samples (ca. 2.0 L, wet weight) were thawed and extracted exhaustively with 2:1 CH_2_Cl_2_/MeOH. After the solvent was evaporated in vacuo, 2.19 g of a crude organic extract was obtained. The extract was then extracted using normal phase Si gel column chromatography using stepwise gradient with increasing polarity of 100% hexanes, 9:1 hexanes/EtOAc, 4:1 hexanes/EtOAc, 3:2 hexanes/EtOAc, 2:3 hexanes/EtOAc, 1:4 hexanes/EtOAc, 100% EtOAc, 9:1 EtOAc/MeOH, 8:2 EtOAc/MeOH. Fractions 8 and 9, eluted with 9:1 EtOAc/MeOH and 8:2 EtOAc/MeOH, respectively, was subjected to solid-phase fractionation on a Sep-Pak C_18_ cartridge (Phenomenex, Torrance, CA, USA) using 100% MeOH to remove pigments. The resulting filtrate was further subjected to semi-preparative RP-HPLC separation (Phenomenex Luna 5μm Phenyl-Hexyl, 250 × 10 mm, 85% MeOH/H_2_O in 60 min at 3.0 mL/min, detected at 210 nm, 230 nm and 290 nm) to yield semi-pure trikoramides B (**1**)–D (**3**). A final purification was achieved using semi-preparative RP-HPLC (Phenomenex Kinetex 5 μm C_18_, 250 × 4.6 mm, 70% MeOH/H_2_O) to yield pure trikoramides B (**1**)−D (**3**) (**1**, 1.1 mg, *t*_R_ = 11.4 min; **2**, 0.3 mg, *t*_R_ = 25.5 min; **3**, 1.2 mg, *t*_R_ = 45.3 min).

### 3.4. Compound Characterization Data

Trikoramide B (**1**): white amorphous solid; ${\left[\alpha \right]}_{D}^{20}$ –108 (c 0.15, MeOH); IR (Nujol) v_max_ 3436, 2953, 1651, 1458, 722 cm^−1^; UV (MeOH) λ_max_ (log ε) 215 (2.91), 295 (2.84), 425 (2.76) nm; ^1^H and ^13^C NMR data (CDCl_3_, 400.13 and 100.61 MHz, respectively), see Table 1 and Supplementary Data; HR-ESI-OrbitrapMS *m*/*z* 1244.7446 [M + H]^+^ (calcd for C_68_H_98_N_11_O_11_, 1244.7441, mass error, 0.401689 ppm).

Trikoramide C (**2**): white amorphous solid; ${\left[\alpha \right]}_{D}^{20}$ –105 (c 0.05, MeOH); IR (Nujol) v_max_ 3436, 2953, 1651, 1458, 722 cm^−1^; UV (MeOH) λ_max_ (log ε) 215 (2.91), 295 (2.84), 425 (2.76) nm; ^1^H and ^13^C NMR data (CDCl_3_, 400.13 and 100.61 MHz, respectively), see Table 2 and Supplementary Data; HR-ESI-OrbitrapMS *m*/*z* 1260.7386 [M + H]^+^ (calcd for C_68_H_98_N_11_O_12_, 1260.7402, mass error, −1.261957 ppm).

Trikoramide D (**3**): white amorphous solid; ${\left[\alpha \right]}_{D}^{20}$ –103 (c 0.07, MeOH); IR (Nujol) v_max_ 3436, 2953, 1651, 1458, 722 cm^−1^; UV (MeOH) λ_max_ (log ε) 215 (2.91), 295 (2.84), 425 (2.76) nm; ^1^H and ^13^C NMR data (CDCl_3_, 400.13 and 100.61 MHz, respectively), see Table 2 and Supplementary Data; HR-ESI-OrbitrapMS *m*/*z* 1339.6805 [M + NH_4_]^+^ (calcd for C_68_H_96_^79^BrN_11_O_11_, 1339.6812, mass error, −0.522512 ppm).

### 3.5. Marfey’s Analysis of Amino Acid Residues in ***1***

Acid hydrolysis of trikoramide B (**1**, 200 μg) was achieved in 1 mL of 1% phenol in 6N HCl placed in a sealed reaction vial purged with N_2_ gas at 110 °C for 5 h. Trace HCl was then removed in vacuo and the resulting hydrolysate was redissolved in 0.1 mL of H_2_O. A 1% solution of L-FDAA (1-fluoro-2,4-dinitrophenyl-5-L-alaninamide) (200 μL) in acetone and 1N NaHCO_3_ (100 μL) was added to the aqueous hydrolysate and the mixture subsequently was heated at 80 °C for 10 min. Once the resulting mixture was cooled to rt, it was sequentially quenched with 2N HCl (100 μL), then dried under vacuum and resuspended in 1:1 H_2_O/CH_3_CN for RP-HPLC analysis. Each HPLC analysis was carried out using a Phenomenex Kinetex C_18_ column (250 × 4.6 mm, 2.6 μm) and an isocratic elution at 40% CH_3_CN–60% 0.05 M trifluoroacetic acid with 0.5 mL/min flow rate over 60 min. The retention times *t*_RL_/*t*_RD_ in min of the L-DAA monoderivatized standards were Pro (10.01/10.67), Val (14.27/20.31), Leu (22.06/34.08), Phe (21.28/30.01), Trp (18.72/22.65). The derivatized hydrolysate peaks of **1** gave retention times at 9.91 min, 14.27 min, 18.85 min, 21.31 min and 22.03 min which corresponded to L-Pro, L-Val, L-Trp, L-Phe and L-Leu, respectively.

### 3.6. MOLT-4 Acute Lymphoblastic Leukemia Cell Line Assay

Assessment of the cytotoxicity of compounds **1** and **3** were carried out using the MTT bioassay based on the MOLT-4 (T lymphoblast; acute lymphoblastic leukemia), cancer cell line over a 3-day procedure. On the first day, **1** and **3** were prepared in a 96-well microtiter plate at 10 mM stock concentration dissolved in 100% DMSO, conducted in triplicate. The mixtures were then added with RPMI media, supplemented with fetal calf serum; and serial diluted to give concentrations of 125, 50, 20, 8 and 3.2 µM. To each of the concentration, 10 µL of the diluted compound **1** was combined with 70 µL of the cancer cells. The plate was incubated for 24 h in a 37 °C, 5% CO_2_ incubator. On day 2, 20 µL of MTT solution were added to each of the wells and incubated for 3 h. Another 100 µL of lysing buffer was added to each well thereafter and incubated overnight. On day 3, the microtiter plate was measured at OD_570_ nm, and the results were tabulated.

### 3.7. Quorum-Sensing Inhibitory Assay

The anti-quorum-sensing bioassay was carried out using *Pseudomonas aeruginosa* PAO1 *lasB-gfp* and *rhlA-gfp* reporter strains. Compounds **1** and **3** were prepared in a 96-well microtiter plate at 10 mM stock concentration dissolved in 100% DMSO, conducted in triplicate. Compounds **1** and **3** were then mixed with ABTGC medium; and serial diluted to give a concentration of 20 µM in the first dilution factor (with 0.2% of DMSO). A total of seven dilution factors, down to 0.3125 µM were done. An overnight culture of PAO1 *lasB-gfp* strain [24], grown in lysogeny broth at 37 °C, 200 rpm, was diluted in ABTGC medium to an optical density of 0.02 at OD_600_ which correspond to 2.5 × 107 CFU/mL. An equal amount of the bacterial suspension was added to reach a final test concentration of 10, 5, 2.5, 1.25, 0.625, 0.3125, and 0.1563 µM. A DMSO control, media control, and culture control were used, and the microtiter plates were incubated at 37 °C in a Tecan Infinite 200 Pro plate reader to measure the cell density (OD_600_) and green fluorescence protein fluorescence (excitation at 483 nm, emission at 535 nm) with 15 min intervals for up to 16 h. Similar procedure was carried out using the PAO1 *rhlA-gfp* biosensor strain.

## 4. Conclusions

Further chemical investigation on the brine shrimp toxic polar VLC fractions obtained from the CH_2_Cl_2_-MeOH (2:1) extracts of the marine cyanobacterium, *S. hydnoides*, from Trikora beach, Bintan, yielded three new cyanobactins trikoramides B (**1**)–D (**3**). Compounds **1**–**3** contained either a hydroxylamine or bromine atom at the Prenyl-Trp residue, which are considered rare post-modifications in the cyanobactin RiPPs pathway. The *C*-prenylated Trp unit has been observed in compounds, such as kawaguchipeptins A–B [25,26], trikoramide A [10] and motobamide A [12] which have all been isolated from cyanobacteria. Trikoramides B and D possessed significant cytotoxicity against MOLT-4 acute lymphoblastic leukemia cell line with an IC_50_ value of 5.2 µM and 4.7 µM, respectively. In addition, the Br-containing trikoramide D exhibited significant dose-dependent response in QS inhibitory assay based on *P. aeruginosa lasB-gfp* and *rhlA-gfp* bioreporter strains with IC_50_ values of 19.6 and 7.3 µM, respectively. Bromination on the prenyl-cyclotryptophan moiety of trikoramide D (**3**) could play a significant role in the QS inhibition in a dose-dependent manner. In fact, previous studies have suggested that the bromination of biologically active compounds increases their effectiveness by enhancing membrane permeability and decreasing metabolic degradation [27]. Due to the relatively large molecular weight of this class of cyanobactins, the addition of bromine may indeed have increased the compound permeability through the membrane and caused significant QS inhibitory activity. A recent study of mono brominated indoles by Kemp et al. sheds light on the effect of bromination on the QS inhibitory activity where the bromination led to the reduction of the IC_50_ by 2 to 13 folds when tested on *E. coli*. [28]. It would therefore be valuable to further study the effect of additional halogenation on this class of cyanobactins on enhancing the QS inhibitory activity against *P. aeruginosa*. In summary, this study reveals the rich bioactivities of secondary metabolites from the marine cyanobacterium, *S. hydnoides*.

## Figures and Tables

**Table 1:** NMR data for trikoramide B (**1**) in CDCl_3_ (^1^H 400 MHz, ^13^C 100 MHz).

Unit	Position	δ_C_, Type	δ_H_ (*J* in Hz)	COSY	HMBC ^a^	NOESY (Selected)
Pro^1^	1	170.2, C				
	2	60.9, CH	4.01, brd (7.9)	3a, 3b	1, 3, 4, 5	2 (Phe)
	3a	31.6, CH_2_	2.19, m	3b, 2, 4	2, 4	*N*H (Leu^3^)
	3b		2.42, m	2, 3a, 4	2, 4	
	4	22.7, CH_2_	1.94, m	3a, 3b, 5a, 5b	2, 3, 5	
	5a	46.7, CH_2_	3.55, m	4, 5b	4	2 (Phe)
	5b		3.67, m	4, 5a	4	
Phe	1	172.5, C				
	2	56.6, CH	3.35, m	3a, 3b, *N*H	1, 3, 4, 5, 9	5a(Pro^1^),2 (Leu^1^)
	3a	34.4, CH_2_	2.27, m	2, 3b	2, 4, 5	
	3b		3.29, m	2, 3a	2, 4, 5	
	4	139.3, C			3, 6, 8	
	5 & 9	130.6, CH	7.13, d (7.5)	6, 8	3, 5, 7, 9	
	6 & 8	127.8, CH	7.23, d (7.4)	5, 7, 9	4, 5, 7, 9	
	7	125.5, CH	7.15, d (7.0)	6, 8	5, 6, 8, 9	
	*N*H		7.48, d (5.3)	2		
Leu^1^	1	172.2, C				
	2	50.1, CH	4.75, m	3a, 3b, *N*H	1, 3, 4	
	3a	41.5, CH_2_	1.67, m	2, 3b, 4	2, 4, 5	
	3b		1.78, m	2, 3a, 4	2, 4, 5	
	4	23.6, CH	1.88, m	3a, 3b, 5, 6	2, 3, 5, 6	
	5	21.5, CH_3_	0.95, d (6.6)	4	2, 3, 4	
	6	23.4, CH_3_	0.85, d (7.4)	4	2, 3, 4	
	*N*H		8.02, brd (7.4)	2		6(Prenyl-Trp)
HydroxylatedPrenyl-Trp	1	173.0, C				
	2	59.5, CH	3.86, dd (9.88, 6.88)	3a, 3b	1, 3	3a, 3b, 2 (Pro^2^)
	3a	37.6, CH_2_	2.23, m	2, 3b	2	
	3b		2.58, m	2, 3a	2	
	4	58.2, C			10, 15, *N*H	
	5	127.2, CH	5.83, brd (15.7)	6	2, 4, 7, 15	
	6	138.8, CH	5.50, brd (15.7)	5	5, 7, 8, 9, 10	*N*H (Leu^1^)
	7	70.7, C			5, 8, 9	
	8	29.3, CH_3_	1.50, s	5, 6	5	
	9	29.4, CH_3_	1.71, s	5,6	5	
	10	82.6, CH	5.42, s		4, 6, 11, 16	3a (Pro^2^), 2 (Leu^1^), *N*H
	11	148.9, C			*N*H, 10, 12, 13, 14	
	12	110.5, CH	6.70, d (7.8)	13	13, 14, 15	
	13	123.5, CH	6.99, d (7.2)	12	12, 14, 15	
	14	119.0, CH	6.75, t (7.4)	15	12, 13, 15	
	15	129.0, CH	7.10, t (7.5)	14	12, 13, 14	
	16	130.9, C			NH, 10	
	*N*H		7.92, brs		4, 11, 16	
Pro^2^	1	171.2, C				
	2	60.4, CH	4.24, brd (8.2)	3a, 3b	3, 4, 5	2 (Prenyl-Trp)
	3a	31.9, CH_2_	2.23, m	2, 3b, 4	2, 4	
	3b		2.42, m	2, 3a, 4	2, 4	
	4	23.8, CH_2_	1.85, m	3a, 3b, 5a, 5b	2, 3, 5	
	5a	47.2, CH_2_	3.53, m	4, 5b	4	
	5b		3.67, m	4, 5a	4	
Val	1	170.5, C				
	2	56.5, CH	4.39, m	3, *N*H	1, 3	5a, 5b (Pro^2^)
	3	30.3, CH	2.21, m	2, 4	2, 4, 5	
	4	18.2, CH_3_	0.87, s	3	3, 5	
	5	19.4, CH_3_	0.88, s	3	3, 4	
	*N*H		7.34, brd (9.7)	2		2 (Pro^3^)
Pro^3^	1	170.8, C				
	2	60.6, CH	4.44, m	3a, 3b	1, 3, 4, 5	
	3a	29.4, CH_2_	1.31, m	2, 3b, 4	2, 4	
	3b		2.17, m	2, 3a, 4	2, 4	
	4	24.9, CH_2_	2.06, m	3a, 3b, 5a, 5b	2, 3, 5	
	5a	40.5, CH_2_	1.54, m	4, 5b	4	2 (Leu^2^)
	5b		1.79, m	4, 5a	4	
Leu^2^	1	172.9, C				
	2	53.6, CH	4.58, m	3a, 3b, *N*H	1, 3, 4	5a, 5b (Pro^3^)
	3a	40.1, CH_2_	1.67, m	2, 3b, 4	2, 4, 5	
	3b		1.85, m	2, 3a, 4	2, 4, 5	
	4	25.3, CH	1.56, m	3a, 3b, 5, 6	2, 3, 5, 6	
	5	23.3, CH_3_	0.87, d (6.8)	4	2, 3, 4	
	6	23.4, CH_3_	0.97, d (6.8)	4	2, 3, 4	
	*N*H		6.79, brd (8.8)	2		H-3a (Pro^4^)
Pro^4^	1	171.8, C				
	2	58.2, CH	3.07, m	3a, 3b	3, 4, 5	
	3a	28.1, CH_2_	1.52, m	2, 3b, 4	2, 4	*N*H (Leu^2^)
	3b		2.11, m	2, 3a, 4	2, 4	
	4	24.6, CH_2_	2.06, m	3a, 3b, 5a, 5b	2, 3, 5	
	5a	47.8, CH_2_	2.83, m	4, 5b	4	
	5b		3.32, m	4, 5a	4	
Leu^3^	1	170.8, C				
	2	49.9, CH	4.42, m	3a, 3b, *N*H	1, 3, 4	
	3a	26.1, CH_2_	1.05, m	2, 3b, 4	2, 4, 5, 6	
	3b		2.20, m	2, 3a, 4	2, 4, 5, 6	
	4	24.9, CH	1.53, m	3a, 3b, 5, 6	2, 3, 5, 6	
	5	20.7, CH_3_	0.91, d (6.4)	4	2, 3, 4	
	6	21.0, CH_3_	1.02, d (6.4)	4	2, 3, 4	
	*N*H		7.41, d (8.4)	2		3a (Pro^1^)

^a^ HMBC correlations optimized for 2/3JCH = 8.0 Hz, are from protons stated to the indicated carbon.

**Table 2:** NMR data of trikoramides C (**2**) and D (**3**) in CDCl_3_ (^1^H 400 MHz, ^13^C 100 MHz).

Unit	Position	Trikoramide C			Trikoramide D		
		δ_C_, Type	δ_H_ (*J* in Hz)	COSY	δ_C_, Type	δ_H_ (*J* in Hz)	COSY
Pro^1^	1	^a^ C			170.2, C		
	2	60.9, CH	4.01, brd (7.9)	3a, 3b	60.8, CH	4.01, brd (8.2)	3a, 3b
	3a	31.6, CH_2_	2.19, m	3b, 2, 4	31.9, CH_2_	2.08, m	3b, 2, 4
	3b		2.42, m	2, 3a, 4		2.48, m	2, 3a, 4
	4	22.7, CH_2_	1.94, m	3a, 3b, 5a, 5b	22.0, CH_2_	1.95, m	3a, 3b, 5a, 5b
	5a	46.7, CH_2_	3.55, m	4, 5b	46.8, CH_2_	3.55, m	4, 5b
	5b		3.67, m	4, 5a		3.67, m	4, 5a
Phe	1	^a^ C			173.2, C		
	2	56.6, CH	3.35, m	3a, 3b, *N*H	56.4, CH	3.34, m	3a, 3b, *N*H
	3a	34.4, CH_2_	2.27, m	2, 3b	33.8, CH_2_	2.89, m	2, 3b
	3b		3.29, m	2, 3a		3.33, m	2, 3a
	4	139.3, C			139.3, C		
	5 & 9	130.6, CH	7.13, d (7.5)	6, 8	130.6, CH	7.15, d (7.4)	6, 8
	6 & 8	127.8, CH	7.23, d (7.4)	5, 7, 9	127.8, CH	7.25, d (7.0)	5, 7, 9
	7	125.5, CH	7.15, d (7.0)	6, 8	125.4, CH	7.14, d (7.4)	6, 8
	*N*H		7.48, d (5.3)	2		7.71, m	2, 5, 9
Leu^1^	1	^a^ C			170.7, C		
	2	49.9, CH	4.42, m	3a, 3b, *N*H	50.2, CH	4.69, m	3a, 3b, *N*H
	3a	41.5, CH_2_	1.67, m	2, 3b, 4	40.3, CH_2_	1.47, m	2, 4
	3b		1.78, m	2, 3a, 4			
	4	23.6, CH	1.88, m	3a, 3b, 5, 6	23.5, CH	1.88, m	3a, 3b, 5, 6
	5	21.5, CH_3_	0.95, d (6.6)	4	21.5, CH_3_	0.99, d (6.6)	4
	6	23.4, CH_3_	0.85, d (7.4)	4	23.3, CH_3_	0.88, d (7.4)	4
	*N*H		8.02, brd (7.4)	2		8.03, brd (8.9)	2
Hydroxylated/Brominated-Prenyl-Trp	1	^a^ C			167.8, C		
	2	59.5, CH	3.94, m	3a, 3b	59.4, CH	3.90, dd (10.3, 6.5)	3a, 3b
	3a	37.6, CH_2_	2.23, m	2, 3b	37.2, CH_2_	2.57, dd (12.4, 7.2)	2, 3b
	3b		2.58, m	2, 3a		2.26, m	2, 3a
	4	^a^ C			59.1, C		
	5	131.2, CH	5.87, brd (16)	6	126.7, CH	5.81, brd (15.6)	6, 8, 9
	6	134.8, CH	5.48, brd (16.4)	5	139.1, CH	5.52, brd (16)	5, 8, 9
	7	70.5, C			70.7, C		
	8	29.3, CH_3_	1.50, s	5, 6	30.3, CH_3_	1.42, s	5, 6
	9	29.4, CH_3_	1.71, s	5,6	29.8, CH_3_	1.28, s	5,6
	10	83.3, CH	5.63, s		81.4, CH	5.42, s	
	11	148.9, C			148.1, C		
	12	110.3, CH	6.70, d (7.8)	13	126.4, CH	7.23, m	13
	13	123.6, CH	6.99, d (7.2)	12	111.4, CH	6.59, d (7.6)	12
	14	119.2, CH	6.75, t (7.4)	15	110.2, C		
	15	129.3, CH	7.10, t (7.5)	14	131.8, CH	7.06, d (1.96)	
	16	130.9, C			131.4, C		
	*N*-OH/*N*H		not obs			8.01, brs	
Pro^2^	1	^a^ C			171.5, C		
	2	60.4, CH	4.24, brd (8.2)	3a, 3b	60.8, CH	4.24, brd (8.5)	3a, 3b
	3a	31.9, CH_2_	2.94, m	2, 3b, 4	31.9, CH_2_	2.23, m	2, 3b, 4
	3b		2.42, m	2, 3a, 4		2.42, m	2, 3a, 4
	4	23.8, CH_2_	1.85, m	3a, 3b, 5a, 5b	23.4, CH_2_	1.85, m	3a, 3b, 5a, 5b
	5a	47.2, CH_2_	3.53, m	4, 5b	47.2, CH_2_	3.53, m	4, 5b
	5b		3.67, m	4, 5a		3.67, m	4, 5a
Val	1	^a^ C			170.5, C		
	2	56.5, CH	4.39, m	3, *N*H	56.7, CH	4.45, m	3, *N*H
	3	30.3, CH	2.21, m	2, 4	30.2, CH	2.21, m	2, 4
	4	18.2, CH_3_	0.87, s	3	18.1, CH_3_	0.87, s	3
	5	19.4, CH_3_	0.88, s	3	19.4, CH_3_	0.88, s	3
	*N*H		7.34, brd (9.7)	2		7.34, brd (9.7)	2
Pro^3^	1	^a^ C			170.8, C		
	2	60.6, CH	4.44, m	3a, 3b	60.6, CH	4.44, m	3a, 3b
	3a	29.4, CH_2_	1.31, m	2, 3b, 4	29.4, CH_2_	1.31, m	2, 3b, 4
	3b		2.17, m	2, 3a, 4		2.17, m	2, 3a, 4
	4	24.9, CH_2_	2.06, m	3a, 3b, 5a, 5b	24.9, CH_2_	2.06, m	3a, 3b, 5a, 5b
	5a	40.5, CH_2_	1.54, m	4, 5b	40.5, CH_2_	1.54, m	4, 5b
	5b		1.79, m	4, 5a		1.79, m	4, 5a
Leu^2^	1	^a^ C			172.9, C		
	2	53.6, CH	4.58, m	3a, 3b, *N*H	53.4, CH	4.57, m	3, *N*H
	3a	40.1, CH_2_	1.67, m	2, 3b, 4	40.1, CH_2_	1.69, m	2, 4
	3b		1.85, m	2, 3a, 4			
	4	25.3, CH	1.56, m	3a, 3b, 5, 6	25.3, CH	1.56, m	3a, 3b, 5, 6
	5	23.3, CH_3_	0.87, d (6.8)	4	23.3, CH_3_	0.87, d (6.8)	4
	6	23.4, CH_3_	0.97, d (6.8)	4	23.4, CH_3_	0.97, d (6.8)	4
	*N*H		6.79, brd (8.8)	2		7.51, brd (8.8)	2
Pro^4^	1	^a^ C			170.3, C		
	2	58.2, CH	3.07, m	3a, 3b	58.2, CH	3.07, m	3a, 3b
	3a	28.1, CH_2_	1.52, m	2, 3b, 4	28.1, CH_2_	1.59, m	2, 3b, 4
	3b		2.11, m	2, 3a, 4		2.11, m	2, 3a, 4
	4	24.6, CH_2_	2.06, m	3a, 3b, 5a, 5b	24.6, CH_2_	2.06, m	3a, 3b, 5a, 5b
	5a	47.8, CH_2_	2.23, m	4, 5b	47.8, CH_2_	2.83, m	4, 5b
	5b		3.32, m	4, 5a		3.32, m	4, 5a
Leu^3^	1	^a^ C			170.7, C		
	2	50.1, CH	4.75, m	3a, 3b, *N*H	50.01, CH	4.43, m	3a, 3b, *N*H
	3a	26.1, CH_2_	1.05, m	2, 3b, 4	26.1, CH_2_	1.05, m	2, 3b, 4
	3b		2.20, m	2, 3a, 4		2.20, m	2, 3a, 4
	4	24.9, CH	1.53, m	3a, 3b, 5, 6	24.9, CH	1.53, m	3a, 3b, 5, 6
	5	20.7, CH_3_	0.91, d (6.4)	4	20.7, CH_3_	0.91, d (6.4)	4
	6	21.0, CH_3_	1.02, d (6.4)	4	21.0, CH_3_	1.02, d (6.4)	4
	*N*H		7.41, d (8.4)	2		6.67, brs	2

^a^ Due to minute quantity of trikoramide C, the ^13^C signals of these quaternary carbons were not discernible to be reported.

**Table 3:** Biological activities of trikoramides A (**4**), B (**1**) and D (**3**).

Compound	IC_50_ Values (μM)		
	MOLT-4	*P. aeruginosa lasB-gfp*	*P. aeruginosa rhlA-gfp*
Trikoramide A	4.8	No DDR*	No DDR*
Trikoramide B	5.2	No DDR*	No DDR*
Trikoramide D	4.7	19.6	7.3

No DDR* = No dose-dependent response observed.

## Supplementary Materials

The following are available online at https://www.mdpi.com/article/10.3390/md19100548/s1, Figures S1–S3: HR mass spectra and MS/MS spectra of trikoramides B–D, Figures S4–S11, S12–S14, S15–S21: 1D and 2D NMR spectra of trikoramides B–D.
