# Supplementary material for: Trikoramides B–D, Bioactive Cyanobactins from the Marine Cyanobacterium Symploca hydnoides
Source: Mar Drugs. 2021 Sep 28;19(10):548. doi: 10.3390/md19100548 (PMC8539366; doi:10.3390/md19100548)
Supplement: Supplementary file 1 [file marinedrugs-19-00548-s001.zip › marinedrugs-1402292-supplementary.pdf]

Supplementary Data

Trikoramides B-D, bioactive cyanobactins from the marine cyanobacterium *Symploca hydnoides*

Ma Yadanar Phyo [1], Teo Min Ben Goh [1], Jun Xian Goh [1] and Lik Tong Tan [1, *]

[1] Natural Sciences and Science Education, National Institute of Education, Nanyang Technological University, 1 Nanyang Walk, Singapore 637616, Singapore

*Corresponding author: Tel: +65 6790 3842; Fax: +65 6896 9414

E-mail address: liktong.tan@nie.edu.sg (L.T. Tan)

Keywords: *Symploca hydnoides*; Microcoleaceae; cyanobactin; trikoramides; cytotoxic; quorum sensing inhibitors

Figure	Title	Page
S1	HR mass spectrum and MS/MS spectrum of trikoramide B (**1**)	3
S2	HR mass spectrum and MS/MS spectrum of trikoramide C (**2**)	4
S3	HR mass spectrum and MS/MS spectrum of trikoramide D (**3**)	5
S4	^{1}H NMR (400 MHz, CDCl3) spectrum of trikoramide B (**1**)	6
S5	^{13}C NMR (100 MHz, CDCl3) spectrum of trikoramide B (**1**)	7
S6	DEPT 90 NMR (100 MHz, CDCl3) spectrum of trikoramide B (**1**)	8
S7	DEPT 135 NMR (100 MHz, CDCl3) spectrum of trikoramide B (**1**)	9
S8	HSQC NMR (100 MHz, 400 MHz, CDCl3) spectrum of trikoramide B (**1**)	10
S9	HMBC NMR (100 MHz, 400 MHz, CDCl3) spectrum of trikoramide B (**1**)	11
S10	COSY NMR (400 MHz, CDCl3) spectrum of trikoramide B (**1**)	12
S11	NOESY NMR (400 MHz, CDCl3) spectrum of trikoramide B (**1**)	13
S12	^{1}H NMR (400 MHz, CDCl3) spectrum of trikoramide C (**2**)	14
S13	COSY NMR (400 MHz, CDCl3) spectrum of trikoramide C (**2**)	15
S14	HSQC NMR (400 MHz, 100 MHz, CDCl3) spectrum of trikoramide C (**2**)	16
S15	^{1}H NMR (400 MHz, CDCl3) spectrum of trikoramide D (**3**)	17
S16	^{13}C NMR (100 MHz, CDCl3) spectrum of trikoramide D (**3**)	18
S17	DEPT 90 NMR (100 MHz, CDCl3) spectrum of trikoramide D (**3**)	19
S18	DEPT 135 NMR (100 MHz, CDCl3) spectrum of trikoramide D (**3**)	20
S19	COSY NMR (400 MHz, CDCl3) spectrum of trikoramide D (**3**)	21
S20	HSQC NMR (100 MHz, 400 MHz, CDCl3) spectrum of trikoramide D (**3**)	22
S21	HMBC NMR (100 MHz, 400 MHz, CDCl3) spectrum of trikoramide D (**3**)	23

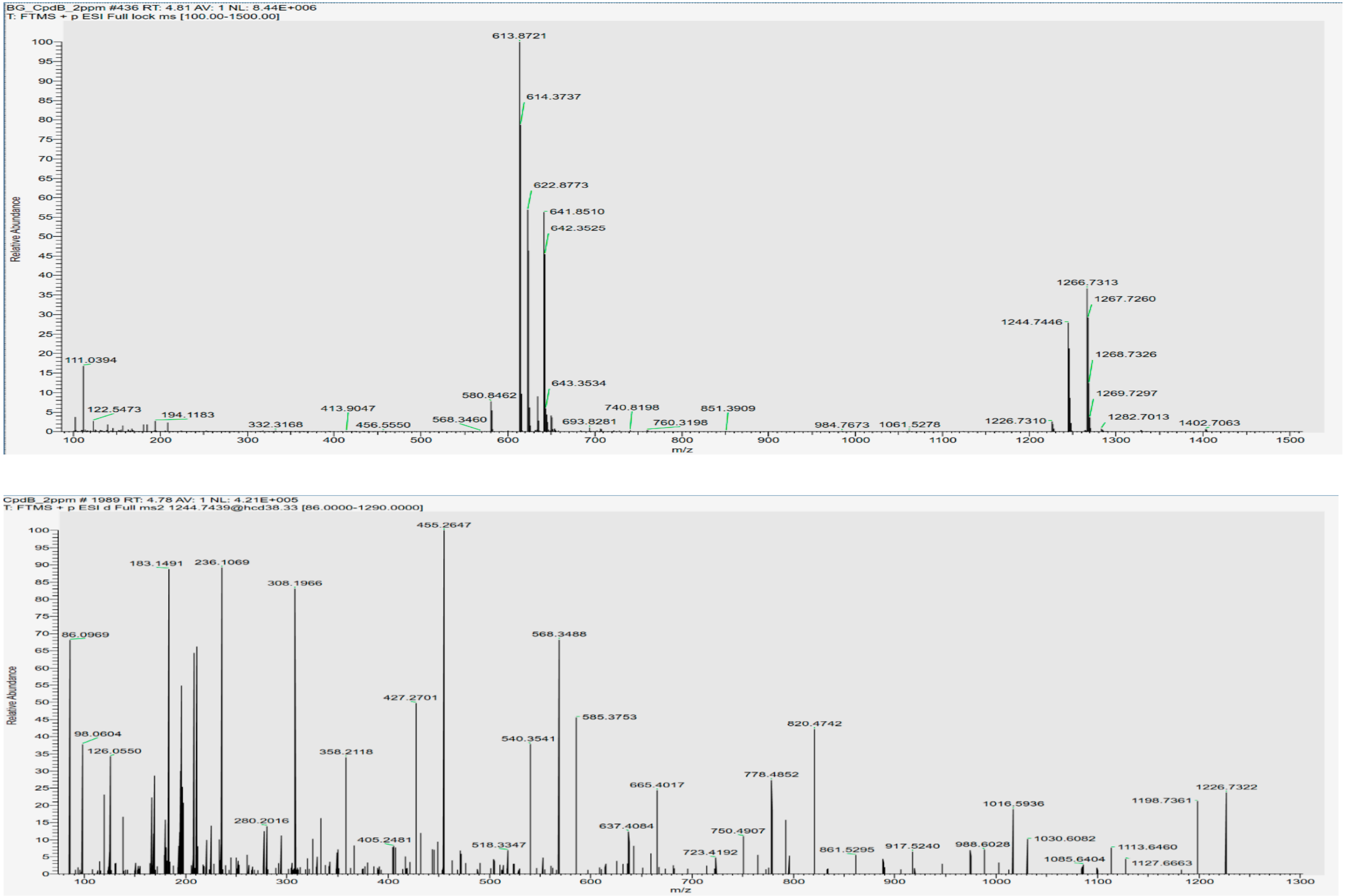

Figure S1. HR mass spectrum and MS/MS spectrum of trikoramide B (**1**).

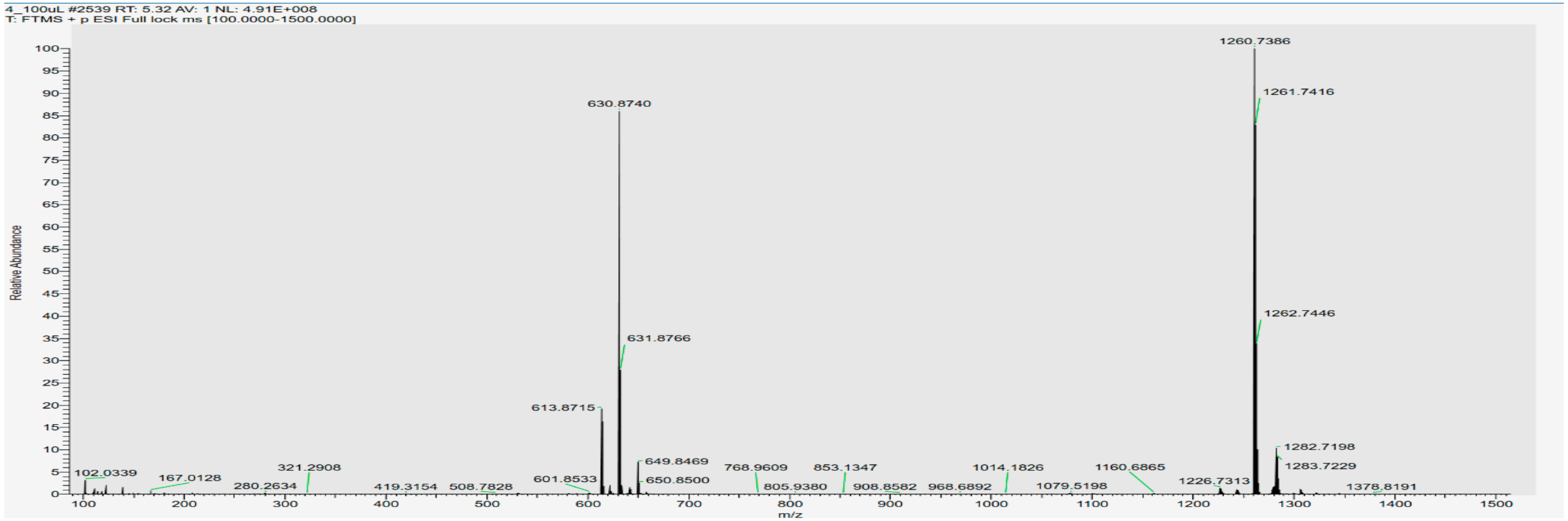

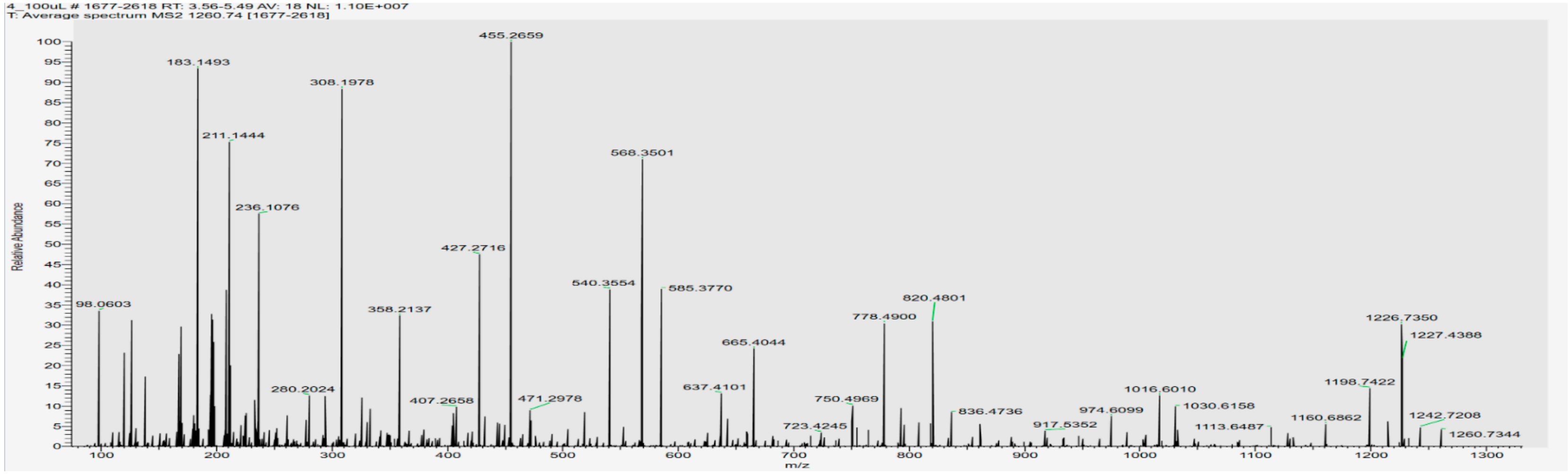

Figure S2. HR mass spectrum and MS/MS spectrum of trikoramide C (**2**).

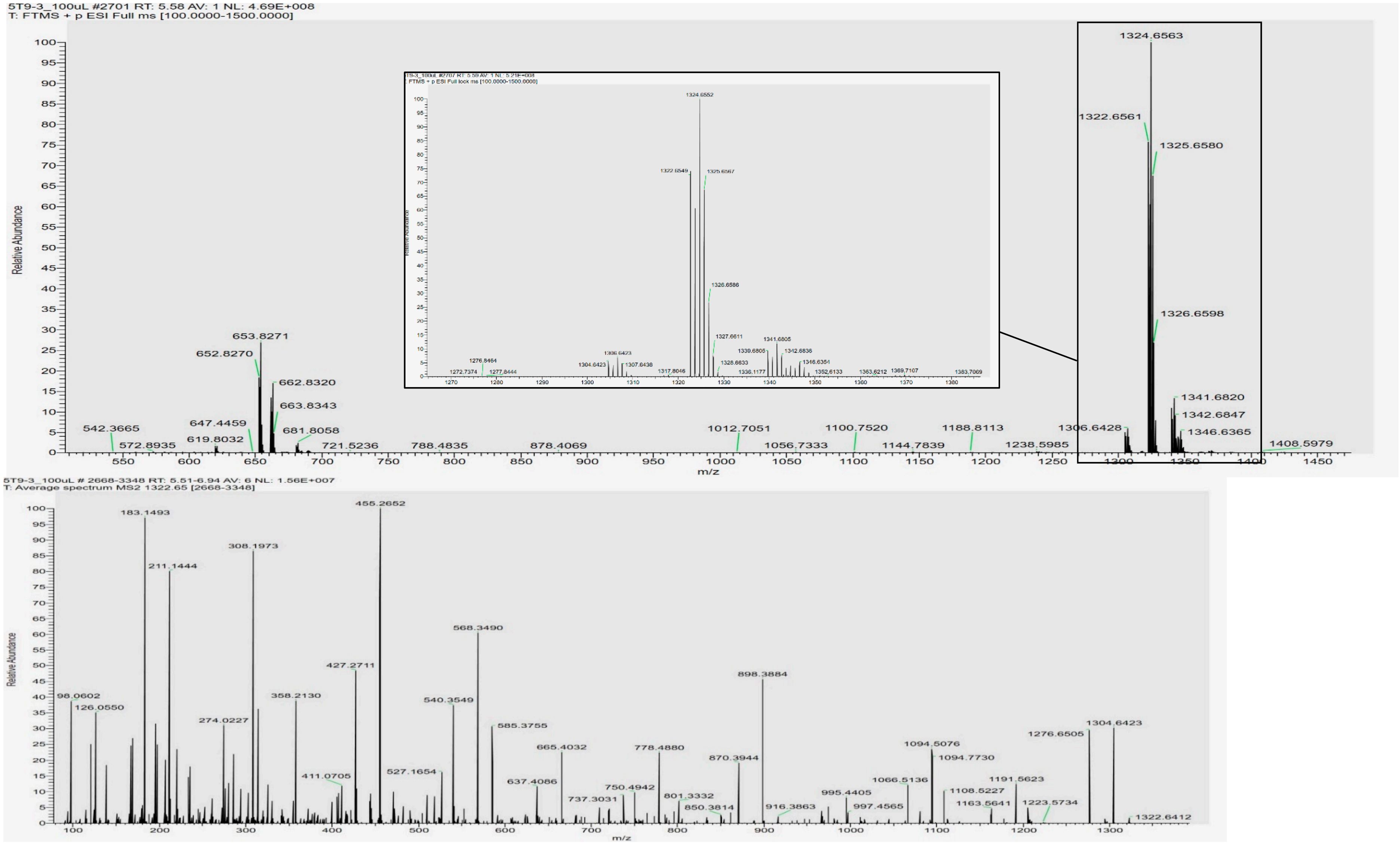

Figure S3. HR mass spectrum and MS/MS spectrum of trikoramide D (**3**).

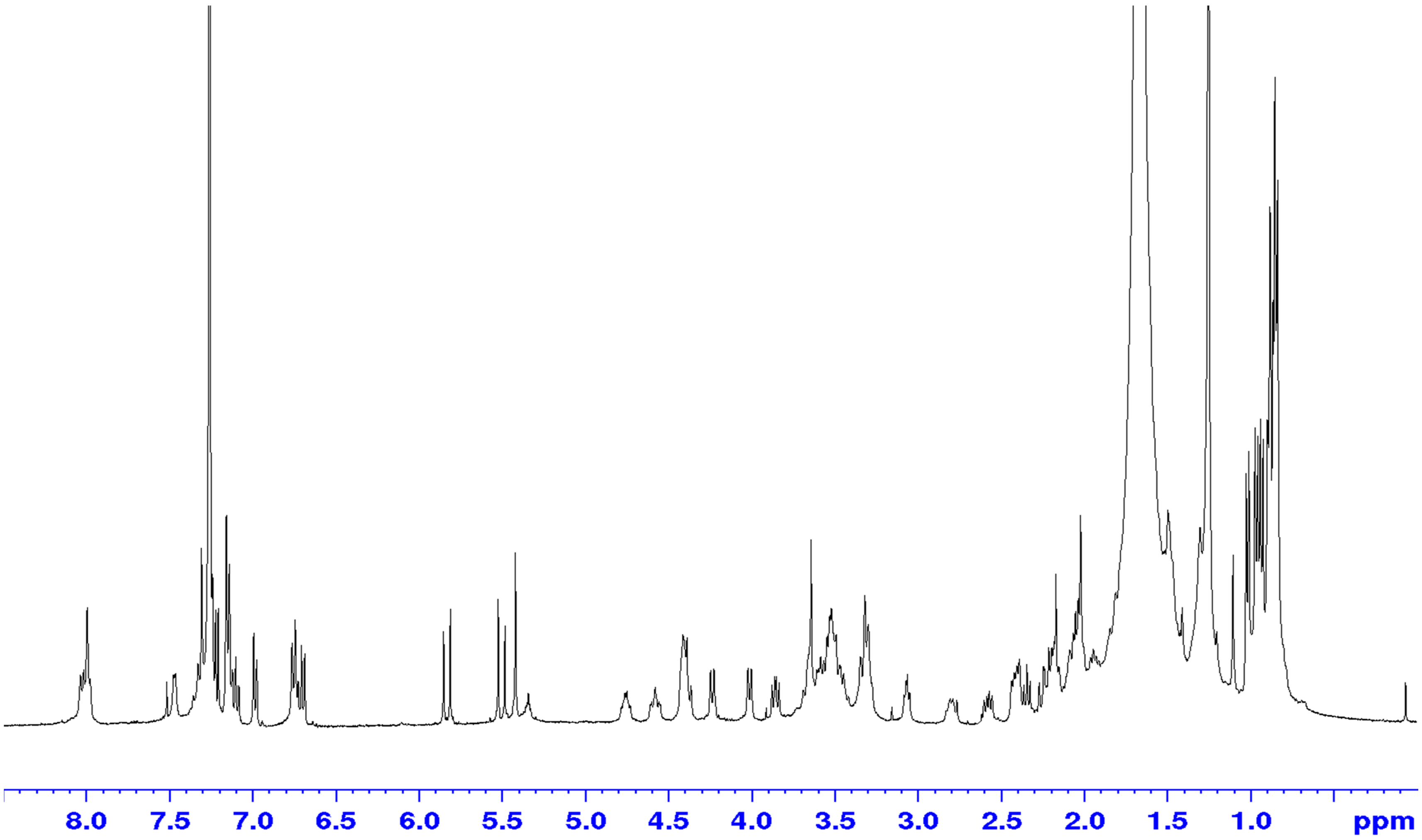

Figure S4. ^{1}H NMR (400 MHz, $CDCl_3$) spectrum of trikoramide B (**1**).

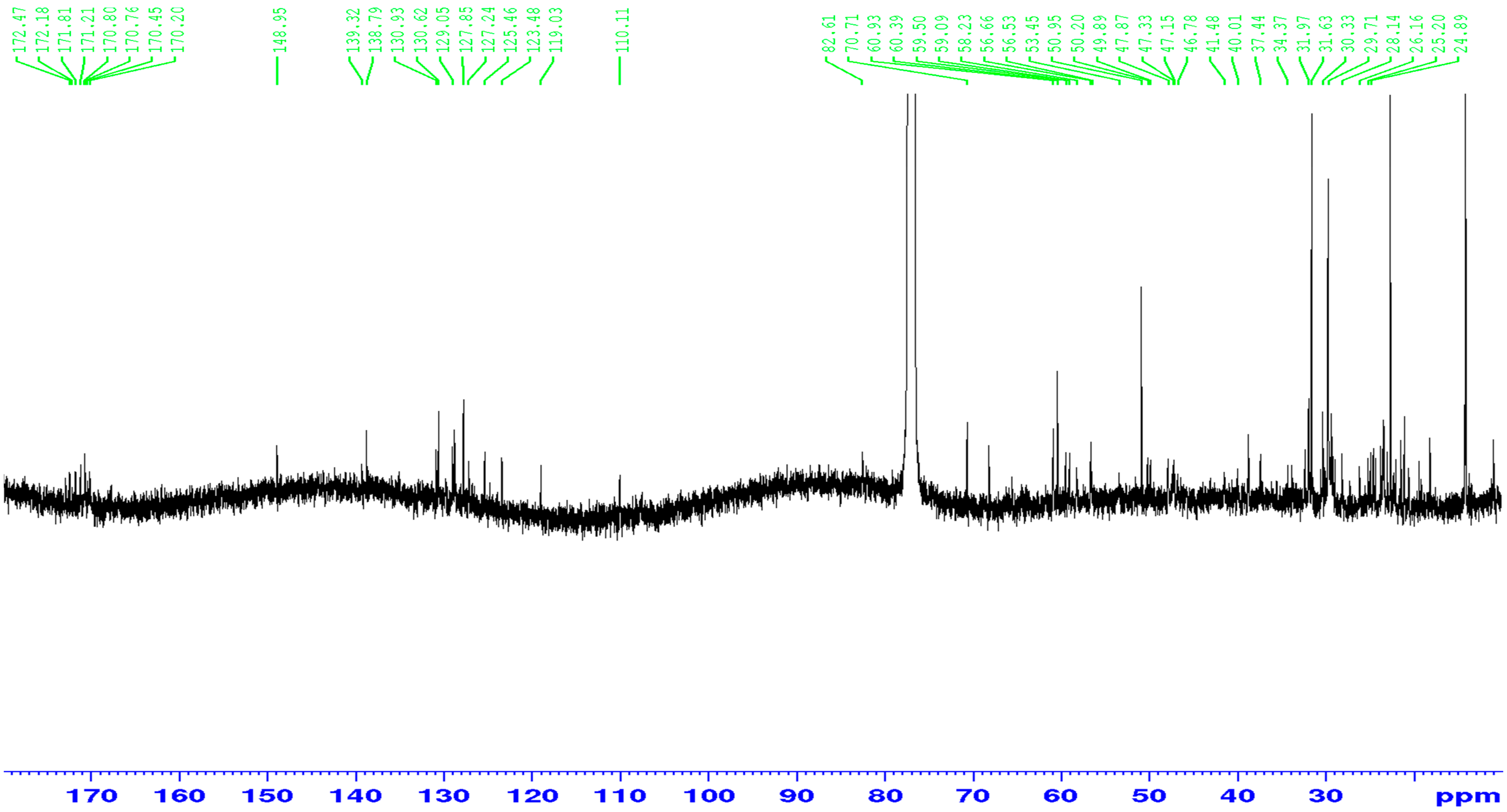

Figure S5. ^{13}C NMR (100 MHz, $CDCl_3$) spectrum of trikoramide B (**1**).

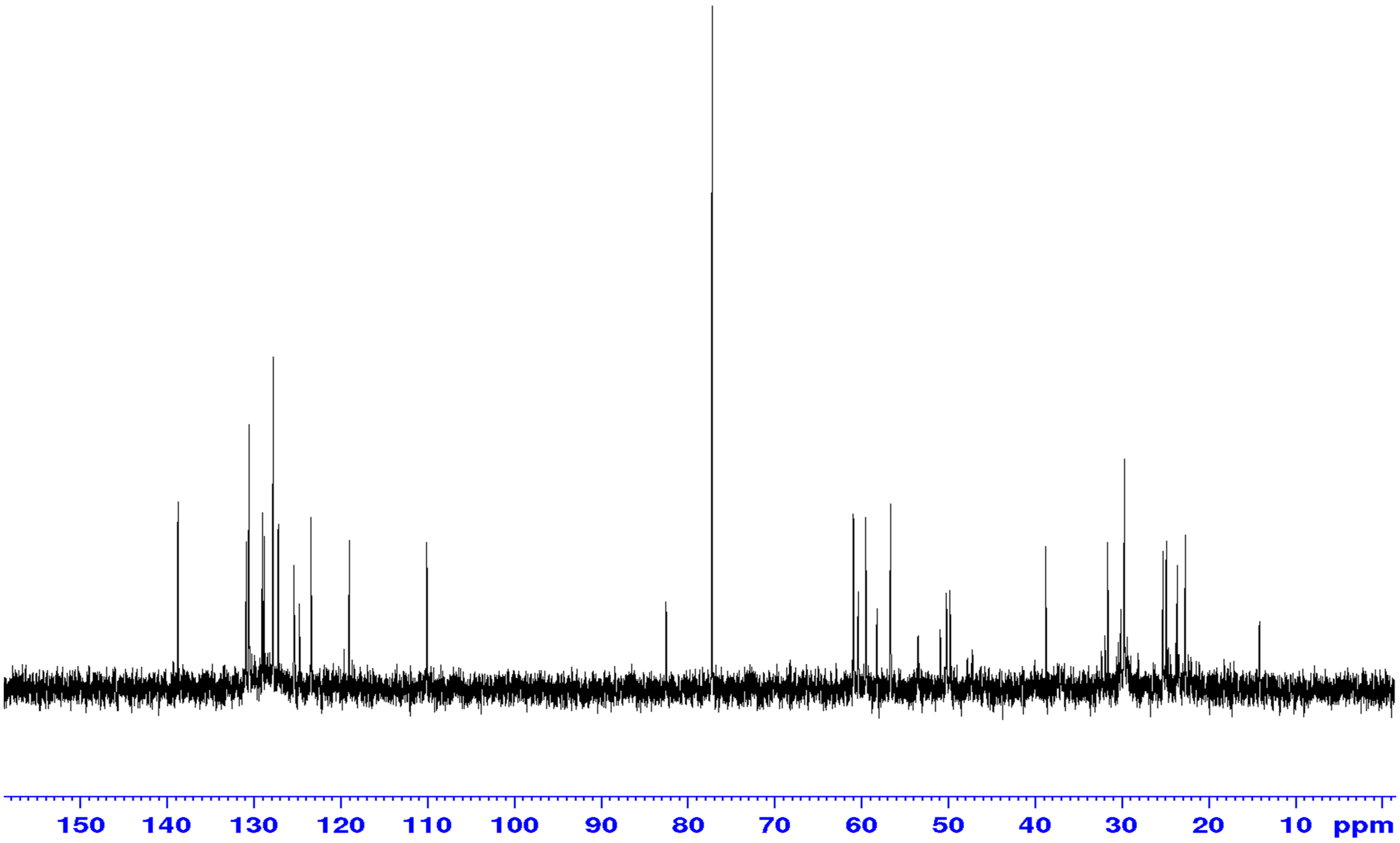

Figure S6. DEPT 90 NMR (100 MHz, $CDCl_3$) spectrum of trikoramide B (**1**).

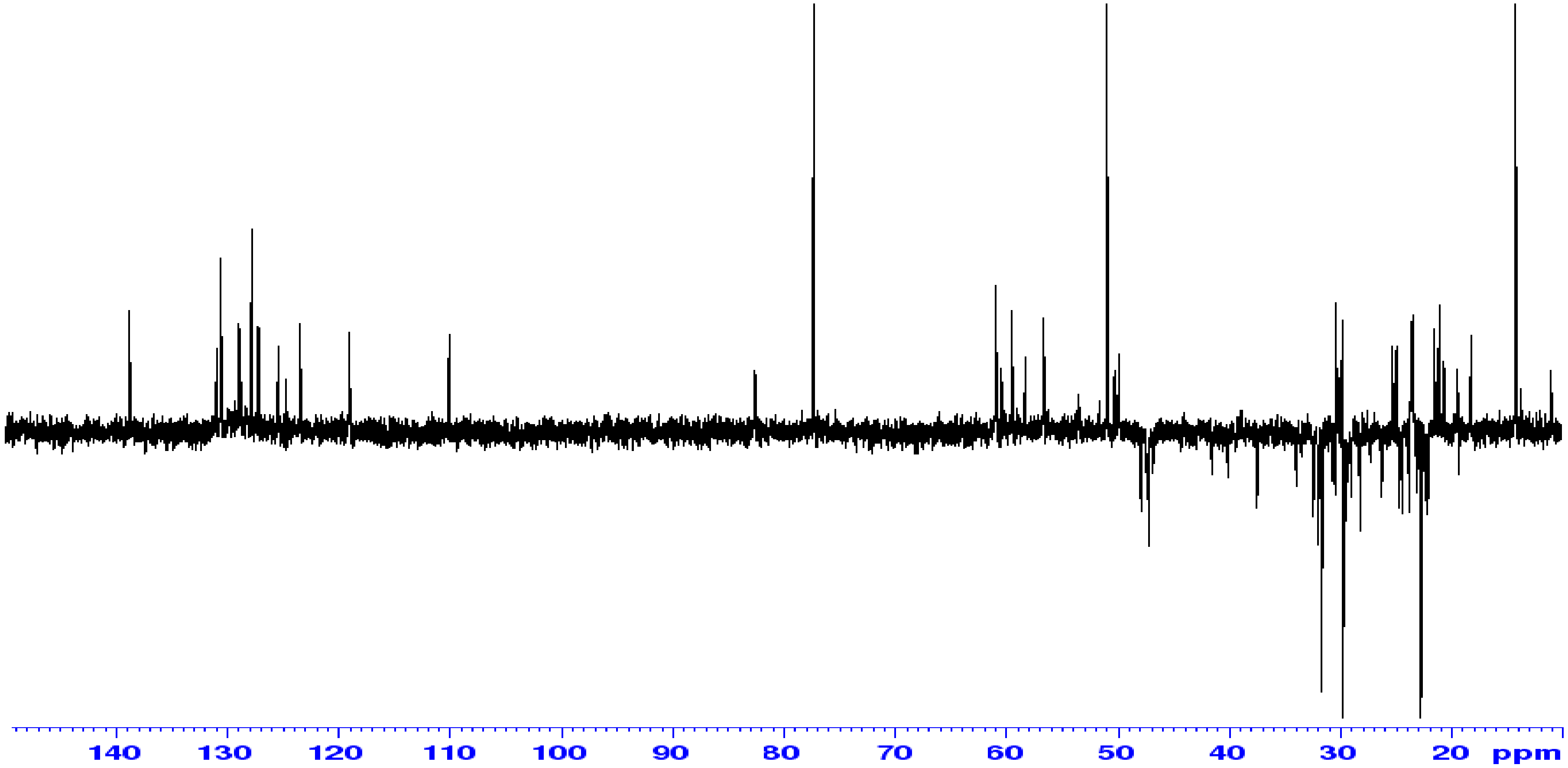

Figure S7. DEPT 135 NMR (100 MHz, $CDCl_3$) spectrum of trikoramide B (**1**).

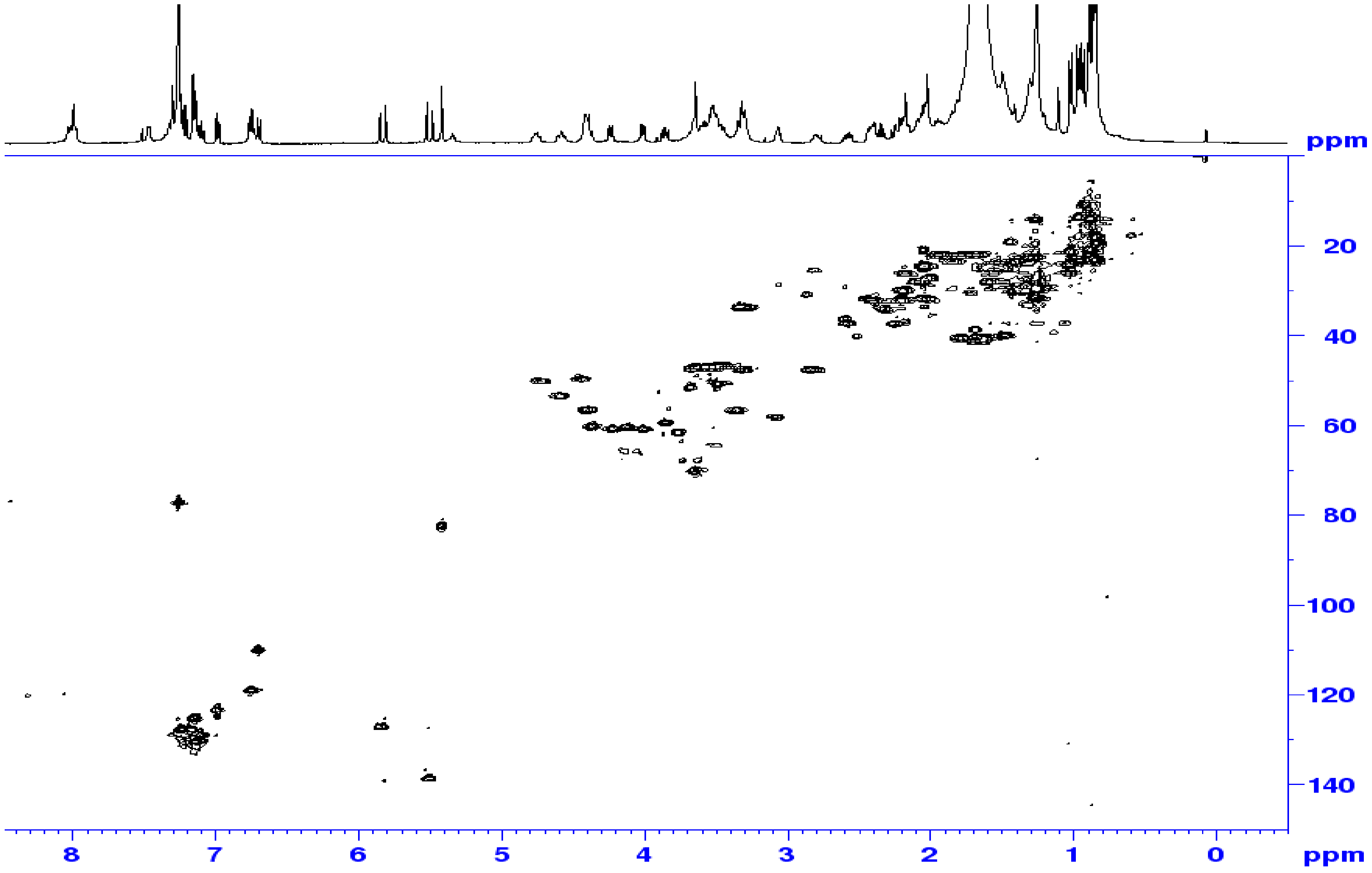

Figure S8. HSQC NMR (100 MHz, 400 MHz, $CDCl_3$) spectrum of trikoramide B (**1**).

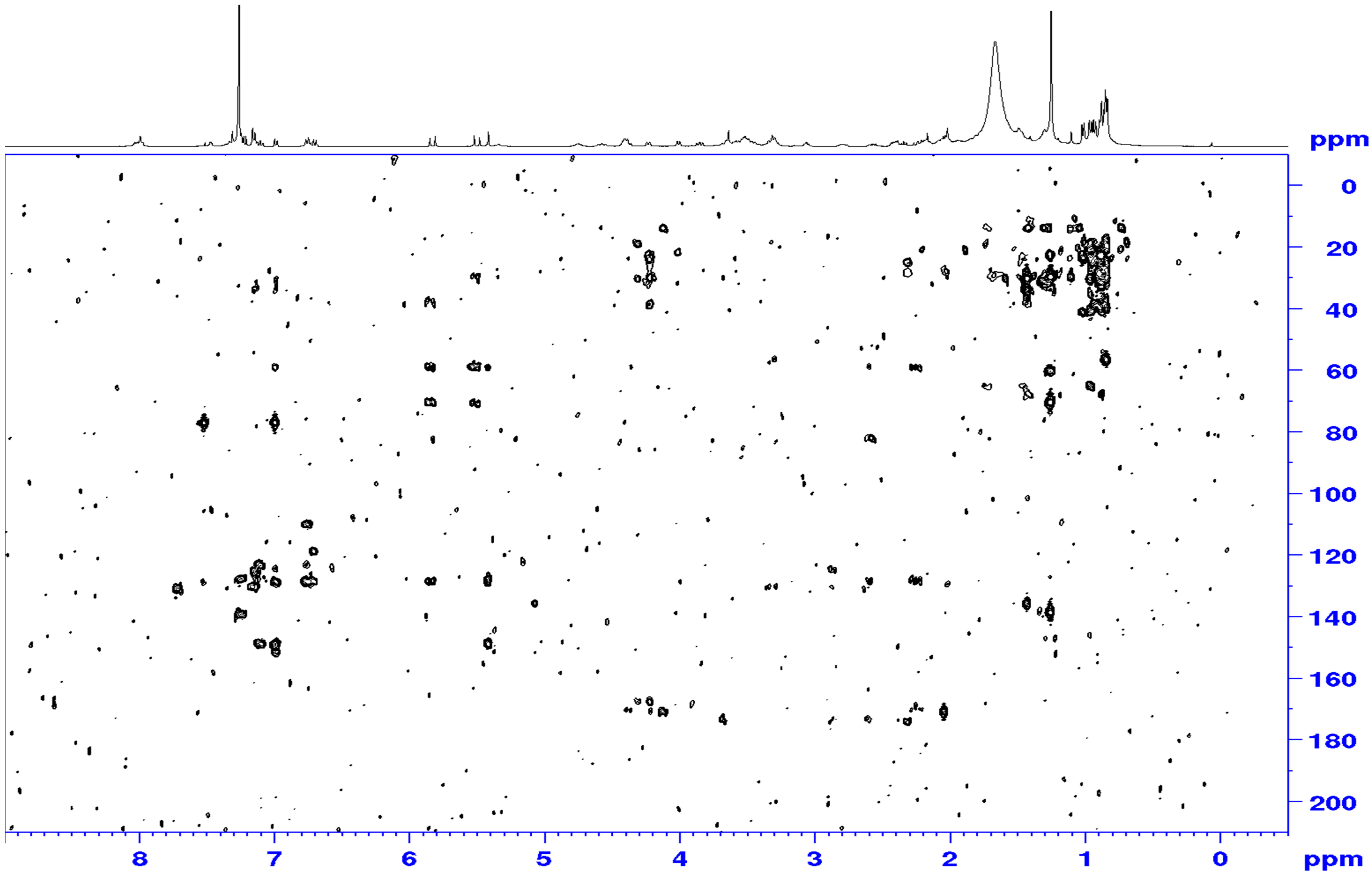

Figure S9. HMBC NMR (100 MHz, 400 MHz, $CDCl_3$) spectrum of trikoramide B (**1**).

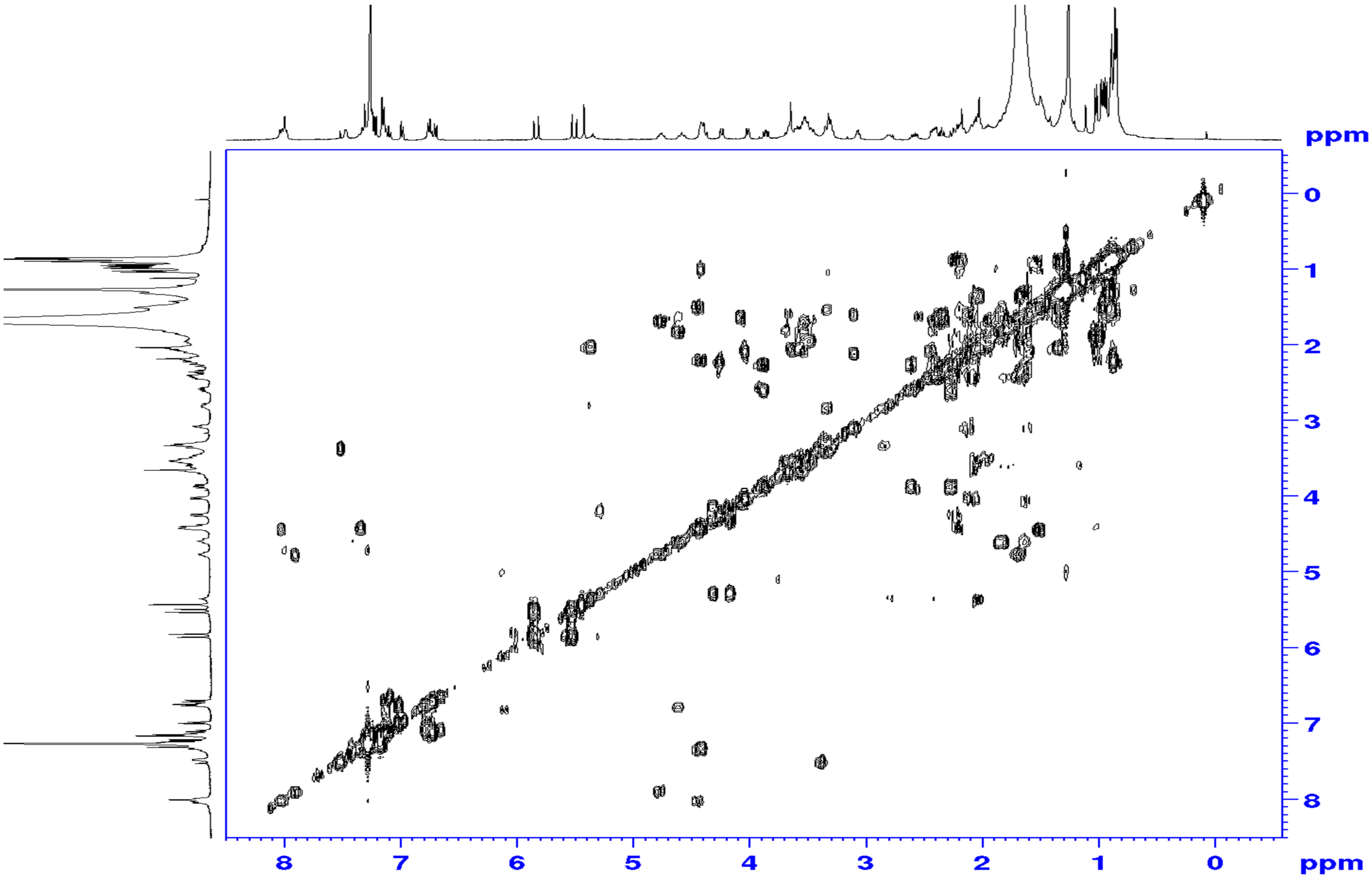

Figure S10. COSY NMR (400 MHz, $CDCl_3$) spectrum of trikoramide B (**1**).

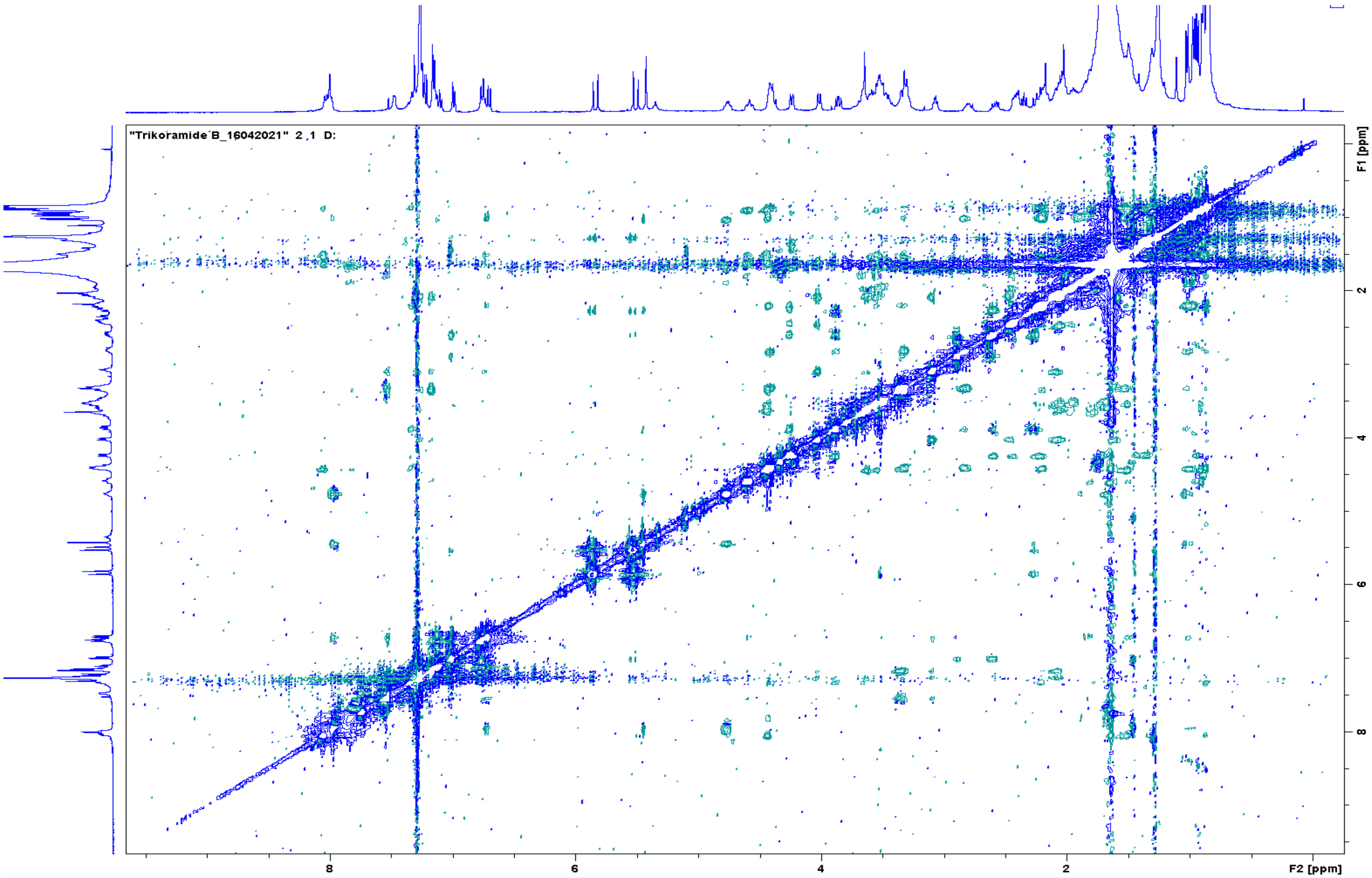

Figure S11. NOESY NMR (400 MHz, $CDCl_3$) spectrum of trikoramide B (**1**).

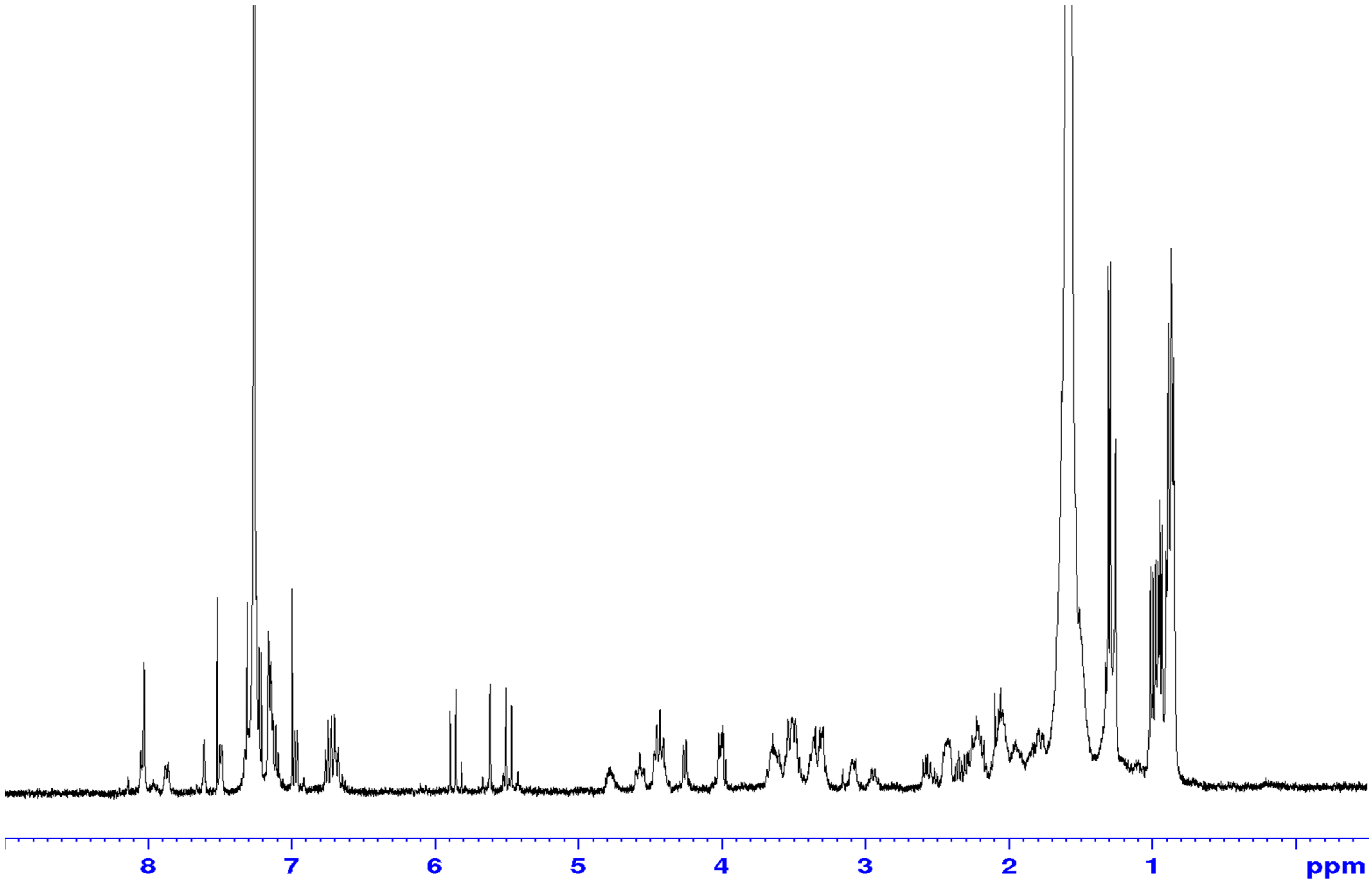

Figure S12. ^{1}H NMR (400 MHz, $CDCl_3$) spectrum of trikoramide C (**2**).

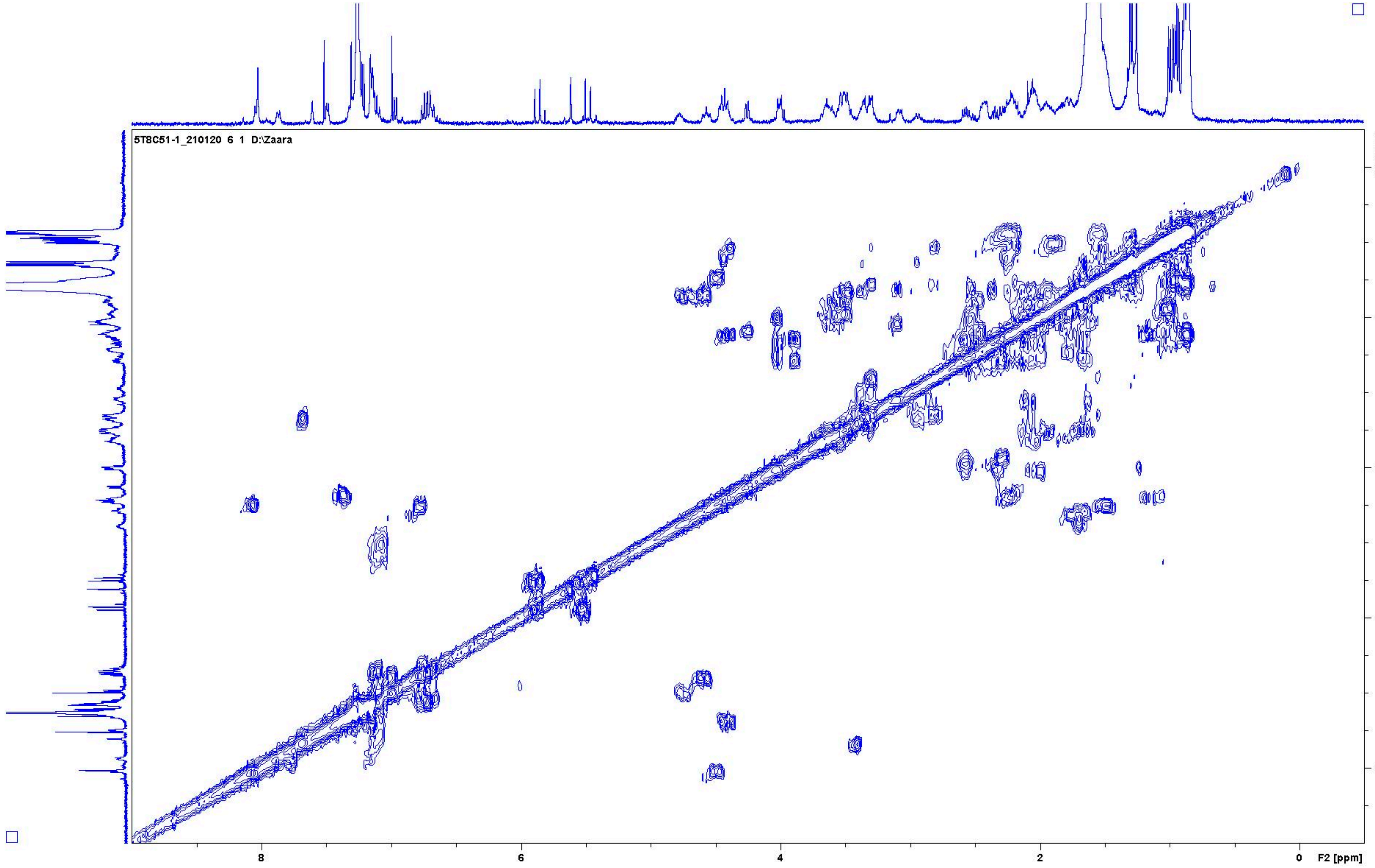

Figure S13. COSY NMR (400 MHz, $CDCl_3$) spectrum of trikoramide C (**2**).

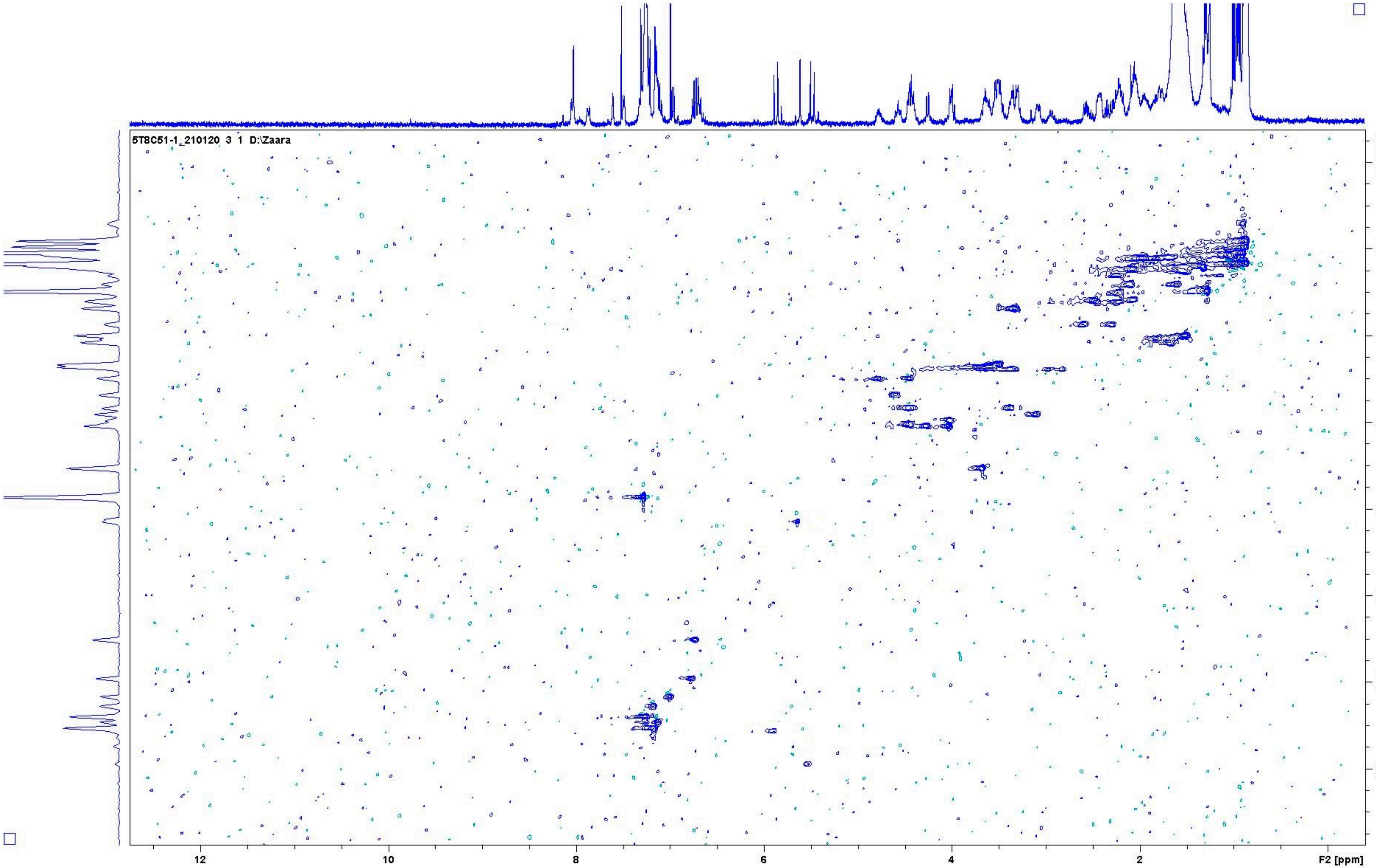

Figure S14. HSQC NMR (400 MHz, 100 MHz, $CDCl_3$) spectrum of trikoramide C (**2**).

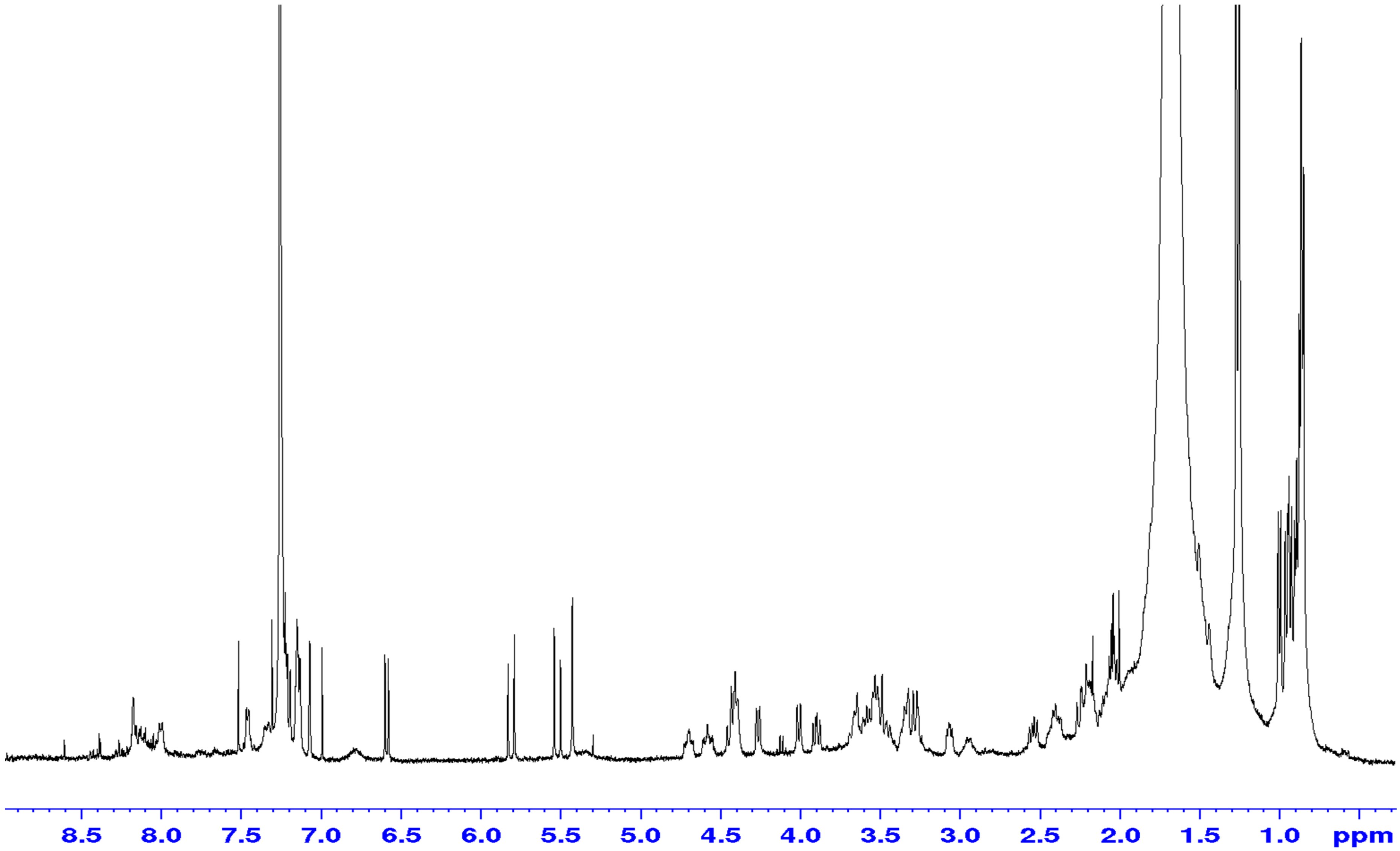

Figure S15. ^{1}H NMR (400 MHz, $CDCl_3$) spectrum of trikoramide D (**3**).

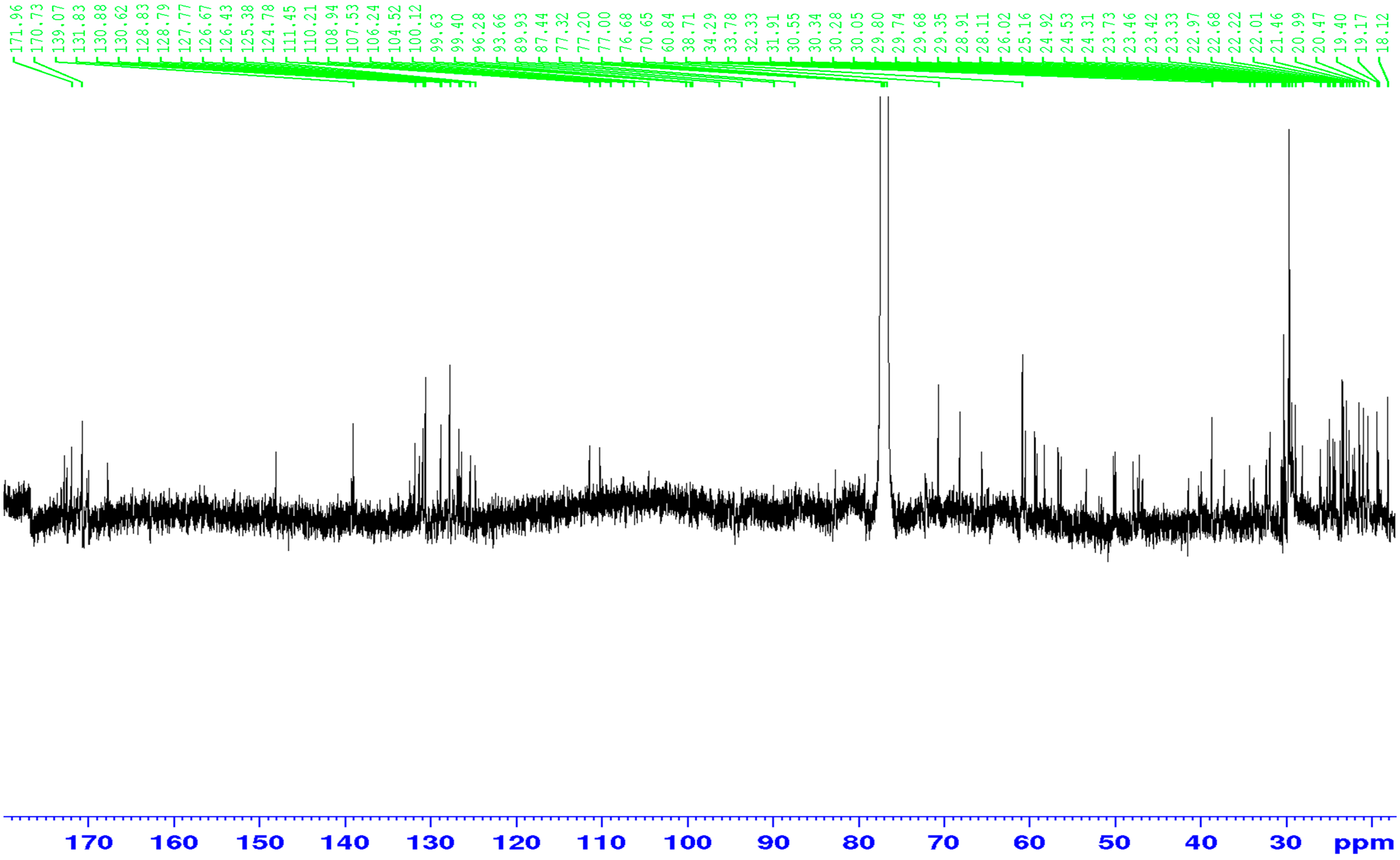

Figure S16. ^{13}C NMR (100 MHz, $CDCl_3$) spectrum of trikoramide D (**3**).

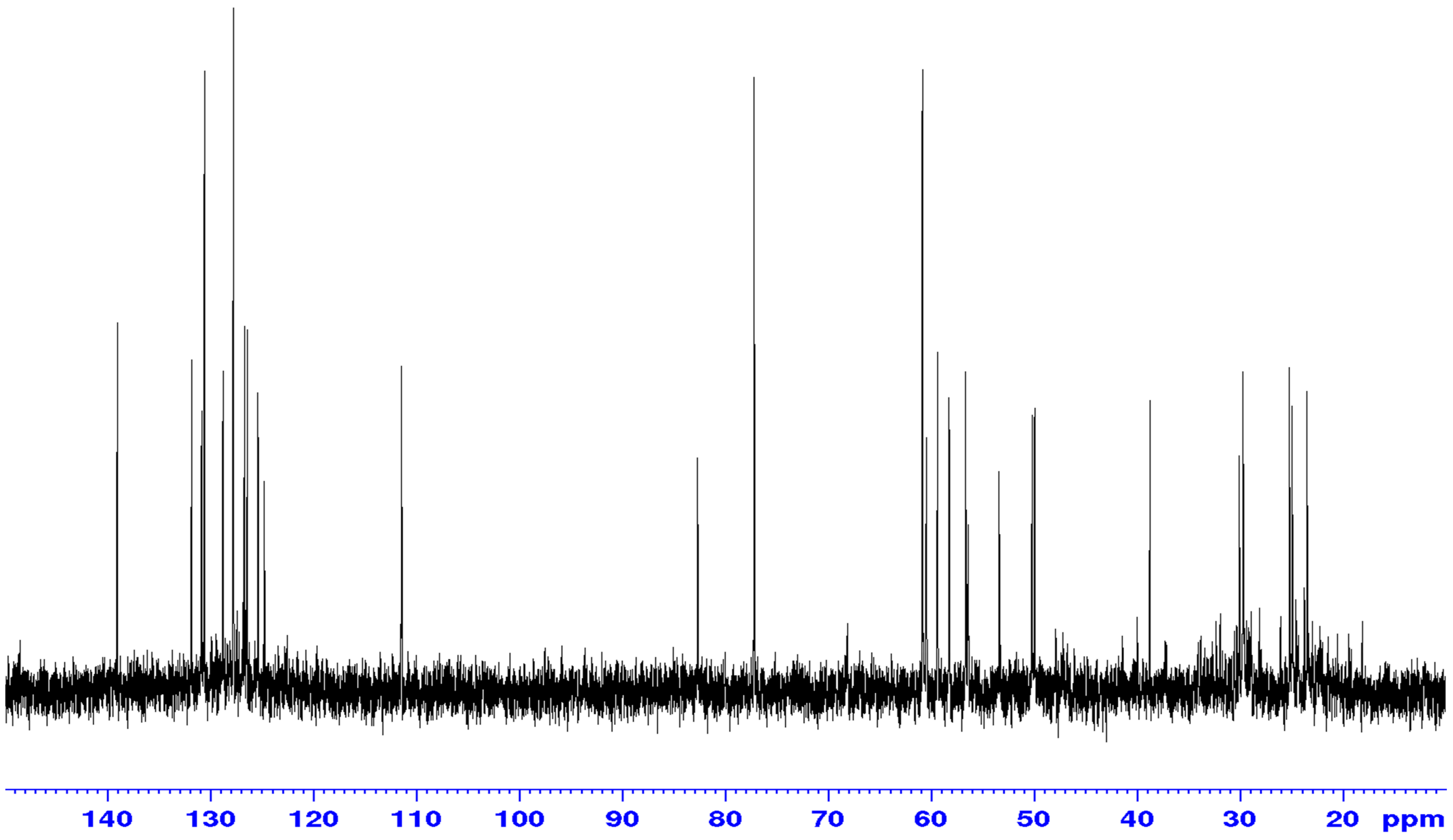

Figure S17. DEPT 90 NMR (100 MHz, $CDCl_3$) spectrum of trikoramide D (**3**).

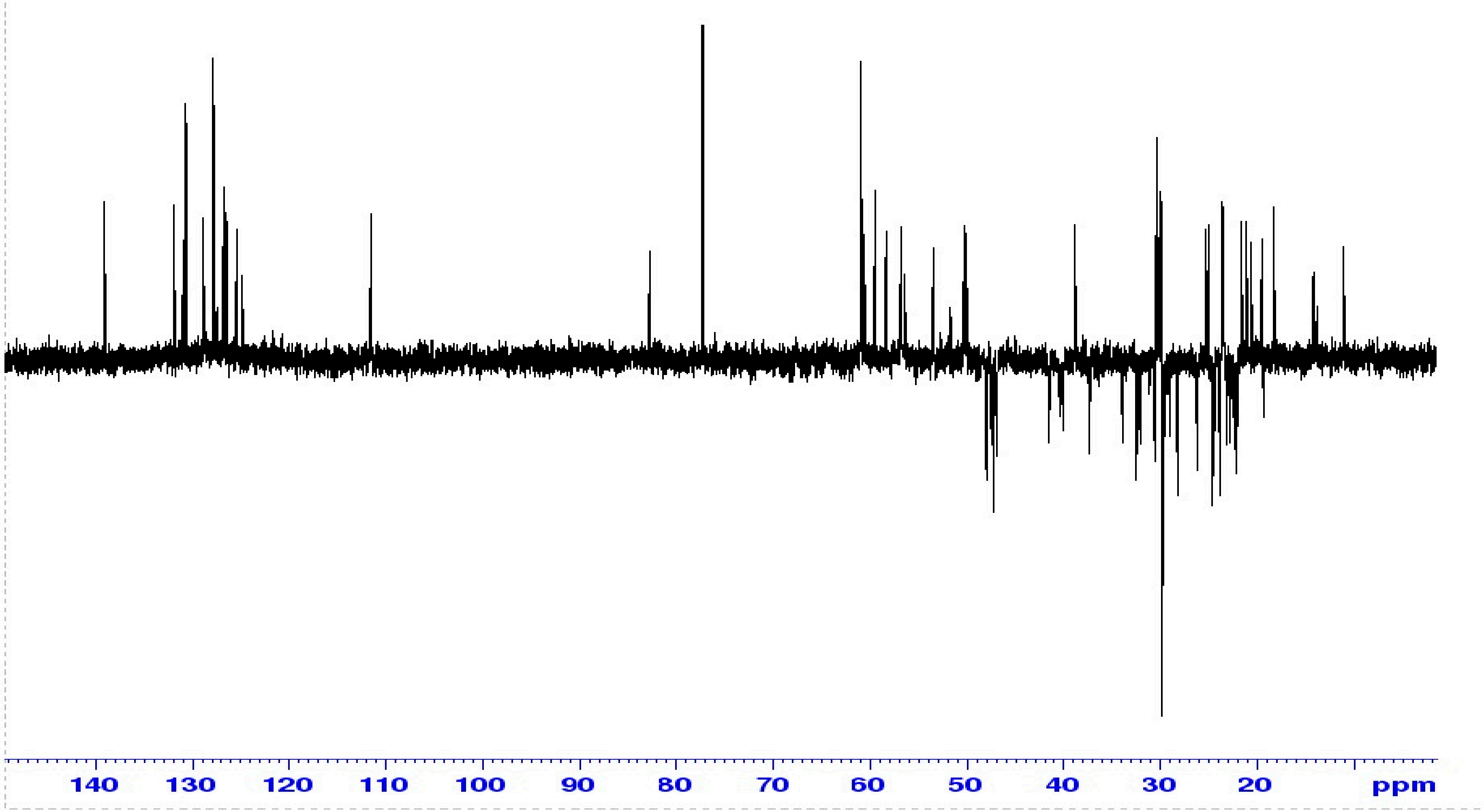

Figure S18. DEPT 135 NMR (100 MHz, $CDCl_3$) spectrum of trikoramide D (**3**).

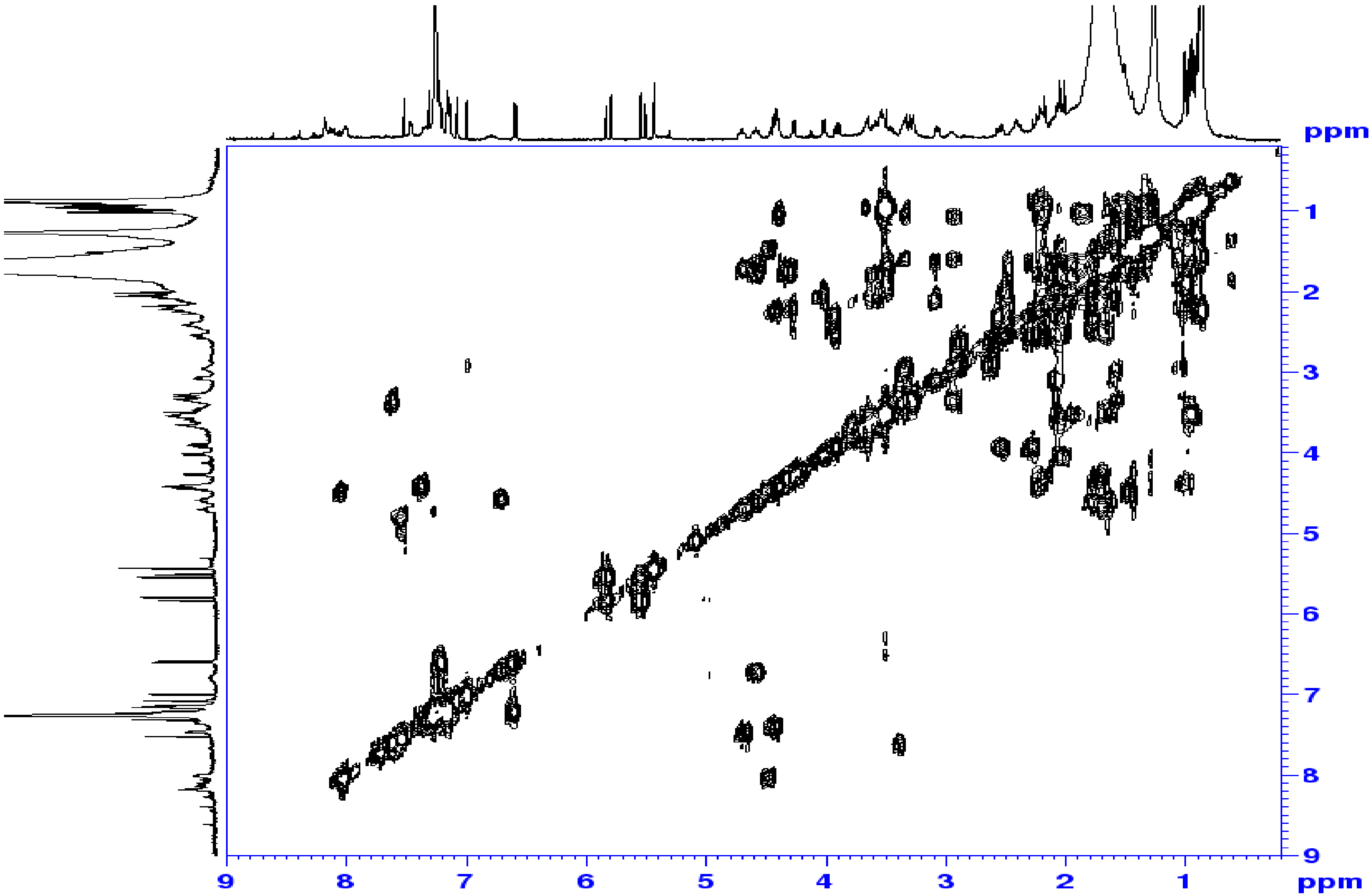

Figure S19. COSY NMR (400 MHz, $CDCl_3$) spectrum of trikoramide D (**3**).

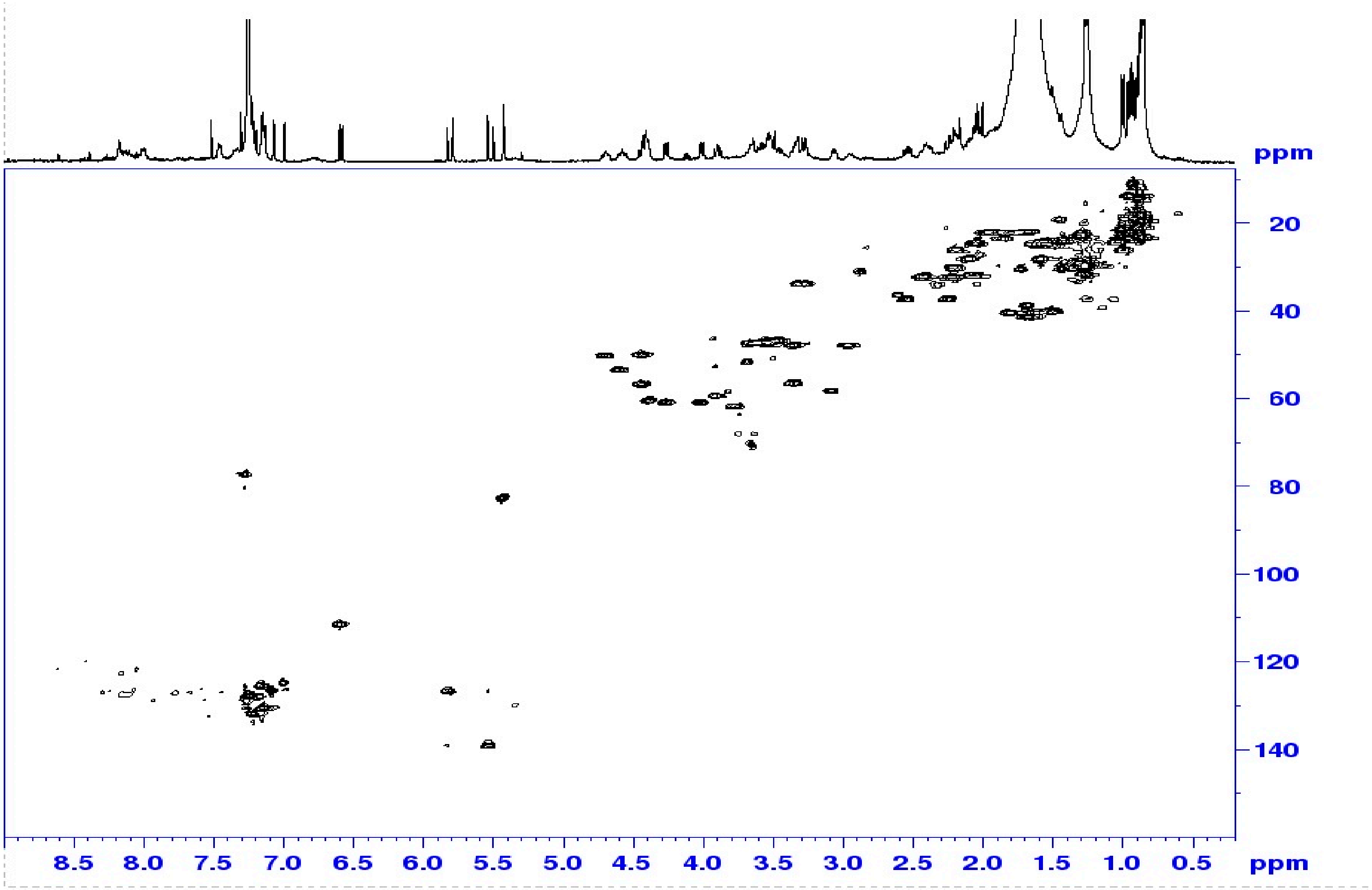

Figure S20. HSQC NMR (100 MHz, 400 MHz, $CDCl_3$) spectrum of trikoramide D (**3**).

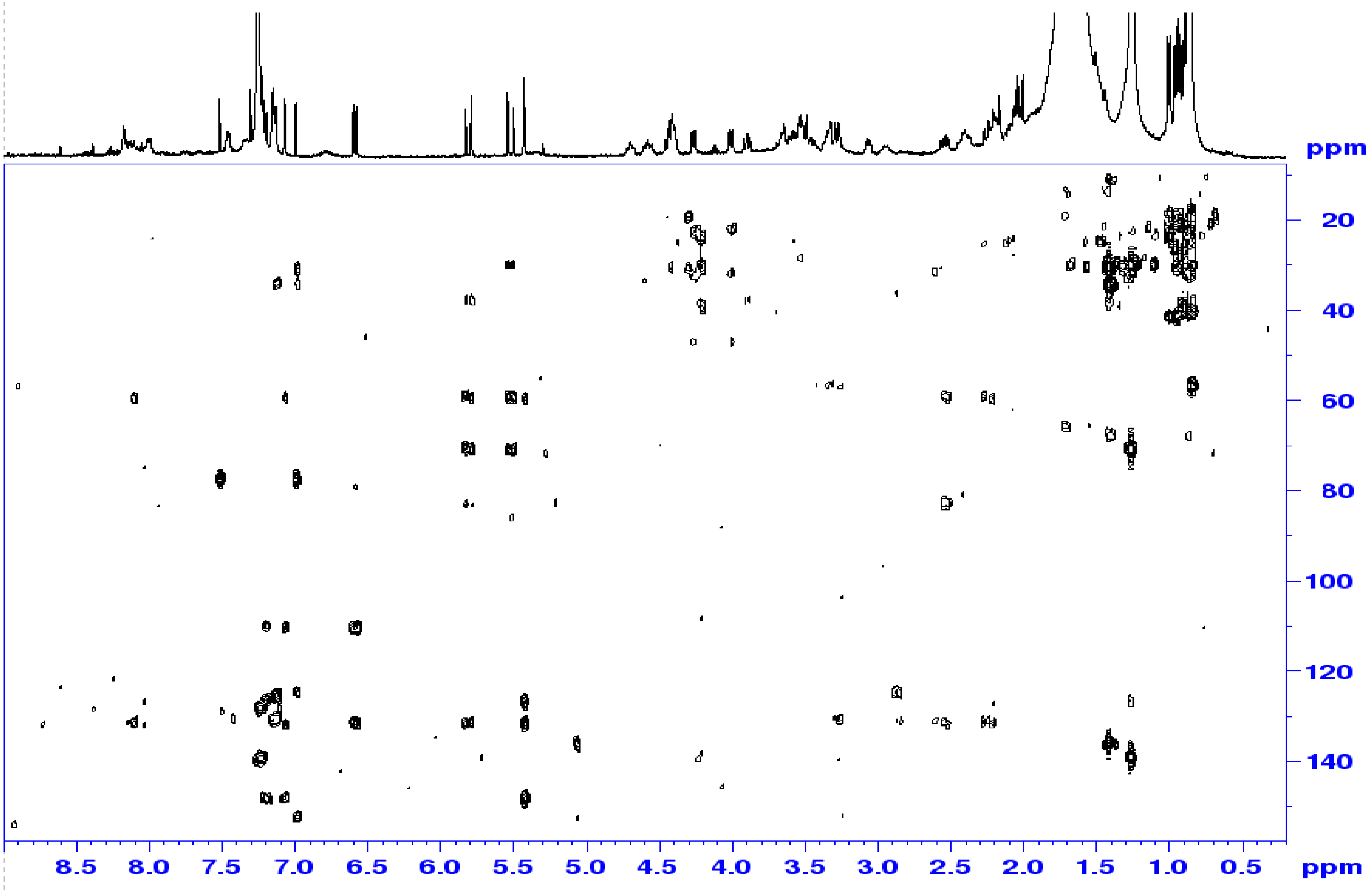

Figure S21. HMBC NMR (100 MHz, 400 MHz, $CDCl_3$) spectrum of trikoramide D (**3**).
